# Superfluidity in topologically nontrivial flat bands

**DOI:** 10.1038/ncomms9944

**Published:** 2015-11-20

**Authors:** Sebastiano Peotta, Päivi Törmä

**Affiliations:** 1 COMP Centre of Excellence, Department of Applied Physics, Aalto University School of Science, FI-00076 Aalto, Finland; 2 Institute for Quantum Electronics, ETH Zürich, CH-8093 Zürich, Switzerland

## Abstract

Topological invariants built from the periodic Bloch functions characterize new phases of matter, such as topological insulators and topological superconductors. The most important topological invariant is the Chern number that explains the quantized conductance of the quantum Hall effect. Here we provide a general result for the superfluid weight *D*_s_ of a multiband superconductor that is applicable to topologically nontrivial bands with nonzero Chern number *C*. We find that the integral over the Brillouin-zone of the quantum metric, an invariant calculated from the Bloch functions, gives the superfluid weight in a flat band, with the bound *D*_s_⩾|*C*|. Thus, even a flat band can carry finite superfluid current, provided the Chern number is nonzero. As an example, we provide *D*_s_ for the time-reversal invariant attractive Harper–Hubbard model that can be experimentally tested in ultracold gases. In general, our results establish that a topologically nontrivial flat band is a promising concept for increasing the critical temperature of the superconducting transition.

## Abstract (web summary)

The critical temperature of superconductors is proportional to the particle coupling energy, but this is different to conventional superfluids where this coupling is small. Here, the authors establish a relation between superconductivity and superfluidity and the topological properties of their band structures.

## Graphical abstract

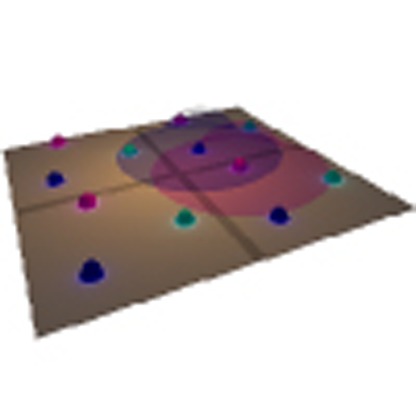

An important result of Bardeen–Cooper–Schrieffer (BCS) theory is the relation 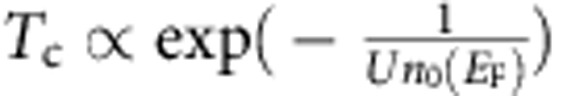, between the critical temperature of the superconducting transition and the microscopic parameters of a superconductor, such as the coupling constant *U* of the effective attractive interaction and the density of states at the Fermi energy *n*_0_(*E*_F_). This result is valid in the limit where the coupling constant *U* is much smaller than the bandwidth, which is roughly given in a tight-binding approximation by the hopping energy *J* between neighbouring atomic orbitals. The BCS formula suggests two ways to increase the critical temperature, namely either to enhance the coupling constant *U* or the density of states *n*_0_(*E*_F_). Whereas the electron–electron attraction parametrized by *U* is the result of complicated many-body physics, not yet well understood in the case of unconventional superconductors, the density of states can be more easily obtained and engineered in a single-particle framework by means of band structure calculations.

The density of states at the Fermi energy *n*_0_(*E*_F_) is maximal for vanishing bandwidth and so is the critical temperature. In this limit, the energy dispersion as a function of lattice quasimomentum *ħ***k** is constant 
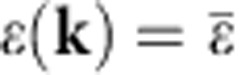
 and the corresponding energy band is called a ‘flat band'. The exponential suppression of the critical temperature disappears in the flat-band limit *U*/*J*≫1 since BCS theory predicts^1^,^2^,^3^,^4^,^5^ *T*_c_∝*Un*_0_(*E*_F_)∝*U*/*J*. This might provide the way to reach the grand goal of room-temperature superconductivity.

A crucial question unaddressed in many works on flat-band superconductors^1^,^2^,^3^,^5^,^6^,^7^,^8^,^9^,^10^,^11^ is whether the superfluid mass density *ρ*_s_, or better, superfluid weight *D*_s_ (see below), is nonzero, leading to the Meissner effect and dissipationless transport^12^,^13^ that define superconductivity. Within the single-band effective Hamiltonian approximation^14^,^15^,^16^, in which only the band dispersion enters, the superfluid weight vanishes (*D*_s_∝*J*) since Cooper pairs localize in the individual lattice sites. Finite superfluid currents can be found in some flat-band systems^17^, but a general theory, connecting the superfluid weight to invariants of the band structure (possibly topological invariants) has not yet been provided. The aim of this work is to answer, at a general level, the crucial question whether superfluidity can exist in a flat band and to explore its possible connections with topological properties of the band.

Using a multiband BCS framework, we show that the superfluid density depends not only on the energy dispersion but also on the Bloch functions of a lattice Hamiltonian. This fact is especially important in the flat-band limit. Moreover, we argue that the superfluid density is subtly affected by the topological invariants encoded in the Bloch functions even in conventional superconductors (not topological)^18^. Topological invariants such as the Chern number *C* are gauge-invariant integer-valued quantities^19^,^20^, which determine the charge and spin conductance and the presence of robust edge states^21^,^22^,^23^. Indeed, the physical picture of localized Cooper pairs is intimately related to the existence of exponentially localized Wannier functions^24^ that can be constructed only if the Chern number *C* is nonzero^25^ (see Fig. 1). Note that the Chern number corresponds to an antisymmetric tensor, the Hall conductance, whereas the superfluid weight is a symmetric one and, if nonzero in a flat band, is an invariant quantity constructed only from the Bloch functions. We find that the superfluid density in a flat band is proportional to a symmetric tensor given by the Brillouin-zone average of a quantity known as the quantum metric^26^,^27^. This tensor is the real part of an invariant matrix 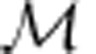, which depends only on the Bloch functions, while the imaginary (antisymmetric) part is the Chern number. By means of the properties of the invariant 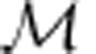, we prove a bound on the superfluid weight that reads *D*_s_⩾|*C*| in appropriate units (see Fig. 1). Moreover, we predict that the superfluid weight is proportional to the coupling constant *D*_s_∝*U* in a flat band. As a concrete application, we derive the superfluid weight in closed form for the Harper–Hubbard model^28^. Using artificial gauge fields, the Harper model has been recently realized with ultracold gases^29^,^30^, which are a good platform to verify our predictions. Our arguments are general and similar results are expected for other flat bands or bands that are only partially flat.

## Results

### Effective lattice Hamiltonian

Our goal is to provide, within a mean-field approximation, a general formula for the superfluid weight of a multiband system that can include topologically nontrivial bands and/or flat bands. A finite supercurrent is associated with a winding of the phase of the superconductor complex order parameter Δ(**r**). In the specific case of a constant current **J**(**q**), the order parameter has the form of a plane wave Δ(**r**)=|Δ|*e*^2i**q**·**r**^ with wavevector 2**q**. The superfluid mass density *ρ*_s_ and superfluid weight *D*_s_ are defined as the change in the free energy density 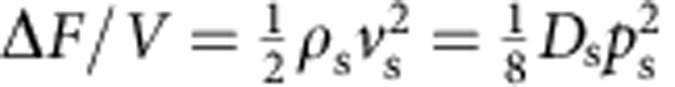 (*V* is the volume in three dimensions, or the area in two dimensions), due to the motion of Cooper pairs with uniform velocity *v*_s_=*ħ*|**q**|/*m* and momentum *p*_s_=2*ħ*|**q**|. In lattice systems, the mass *m* is not a well-defined concept, and it is better to use the superfluid weight^12^,^13^ *D*_s_.

A computationally convenient definition of the superfluid weight is in terms of the grand potential Ω(*T*, *μ*, Δ, **q**) (see ref. 31 and Supplementary Note 1)


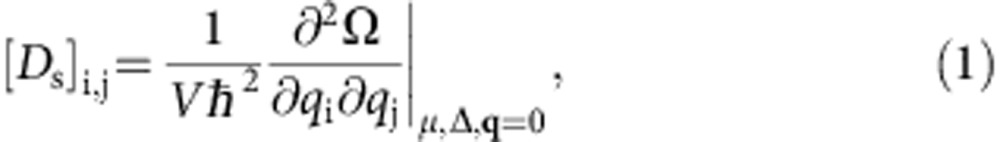


where i,j=*x*, *y*, *z* are spatial indices. In anisotropic and time-reversal invariant systems, the superfluid weight is given by a symmetric tensor [*D*_s_]_i,j_ (the notation [*M*]_i,j_ for the elements of a matrix *M*, with i,j not necessarily spatial indices, is used throughout the article).

In calculating the superfluid weight, we proceed in the following way. (1) The supercurrent wavevector **q** is introduced in the Hamiltonian in a way that is rigorous for topologically nontrivial bands: a multiband approach is used. (2) The kinetic Hamiltonian is Fourier transformed, which defines the band dispersions and the Bloch functions. (3) A mean-field approximation is done by introducing a Bogoliubov-de Gennes (BdG) Hamiltonian. (4) The BdG Hamiltonian is diagonalized to provide a convenient expression for the grand potential. (5) Supercurrent and superfluid weight are obtained as derivatives of the grand potential with respect to **q**: the results are given in terms of the band dispersions and the Bloch functions. (6) The results are connected to topological properties of the system.

Some care is needed to introduce the wavevector **q** in the Hamiltonian in a proper way. By a suitable gauge transformation, it is possible to constrain the complex order parameter Δ(**r**) to be real and have the same translational symmetry as the underlying lattice, whereas the wavevector **q** appears in the kinetic term of the lattice Hamiltonian


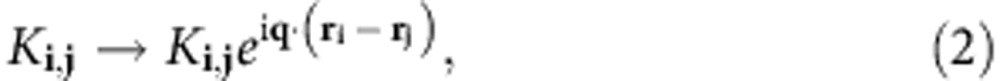


where the matrix elements *K*_**i**,**j**_∝*J* are hopping amplitudes between lattice sites. If the wavevector **q** is identified with a constant external vector potential **A** according to **q**=*q***A**/*ħ*, then equation (2) becomes the usual Peierls substitution.

The Peierls substitution is an approximation valid only if the basis states of the lattice Hamiltonian are well localized^15^,^16^, and ideally they should be exponentially localized Wannier functions^24^. Since bands with a nonzero Chern number do not allow exponentially localized Wannier functions^25^, we use a multiband approach that can circumvent this problem. We consider a subset of bands, which we call 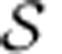, well-separated from other bands by band gaps (a composite band)^24^ such that the Chern number (or numbers) of the composite band is zero. By linear superposition of Bloch functions of all the bands in 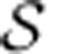, it is possible to construct exponentially localized Wannier functions. For the notation and the definition of Wannier functions, see Fig. 2 and Supplementary Note 2.

In the basis of Wannier functions, the effective lattice Hamiltonian for the composite band reads (the derivation is known^14^, but for convenience we repeat it in Supplementary Note 2)


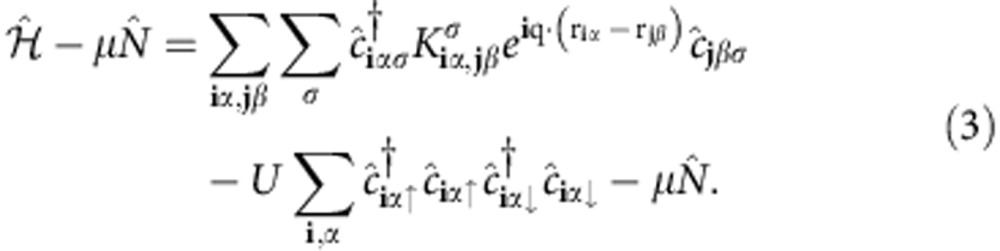


The 
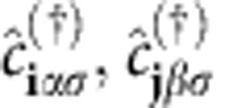
 are annihilation (creation) operators for the orbitals (Wannier functions) labelled by **i***α* and **j***β* (Fig. 2a) and spin σ, μ the chemical potential, 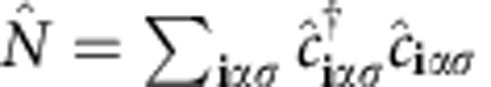 the particle number operator, and we consider the specific case of an attractive Hubbard interaction (*U*>0). The Peierls substitution has been used, properly generalized to the multiband case (see Fig. 2a for the definition of **r**_**i***α*_). For **q**=0, the Hamiltonian is invariant under time-reversal symmetry (TRS) since 
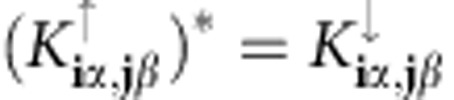
 and invariant under spin rotation around the *z* axis, but in general 
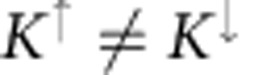
.

Diagonalization of the Fourier transform of the hopping matrix in equation (3) gives the band structure (see Derivation of the Bogoliubov-de Gennes Hamiltonian in Methods)


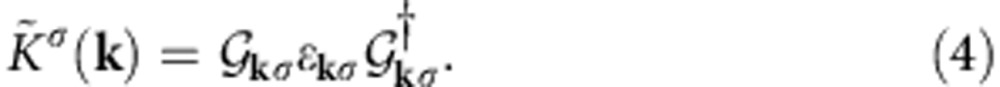


Here *ɛ*_**k***σ*_=diag(*ɛ*_*n***k***σ*_) is a diagonal matrix composed of the dispersions *ɛ*_*n***k***σ*_ of each band (*n* labels a single band belonging to the composite band), while the *n*-th column of the unitary matrix 
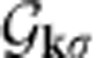
 is the Bloch function 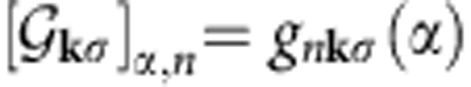 of the *n*-th band. TRS implies that *ɛ*_**k**↑_=*ɛ*_−**k**↓_ and 
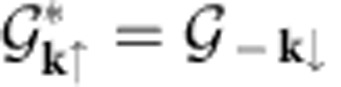
.

### BCS theory and superfluid weight in a multiband system

The idea of superconductivity in multiband systems dates back to 1959 (ref. 32). The first superconductor for which multiband effects are indeed measurable is magnesium diboride (MgB_2_), discovered as recently as^33^,^34^ 2001. However, to the best of our knowledge, a general and consistent theory for the superfluid weight in a multiband system, in particular for topologically nontrivial flat bands, has not yet been worked out.

In the following, we develop the theory of the superfluid weight in a multiband system within the framework of BCS theory, namely, we use a mean-field decoupling of the interaction term


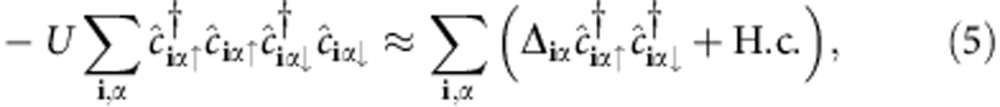


where 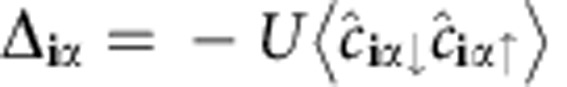. Furthermore, we choose a gauge where the gap function preserves the discrete translational symmetry of the lattice, which means Δ_**i***α*_=Δ_*α*_. Thus the pairing terms are diagonal in momentum space 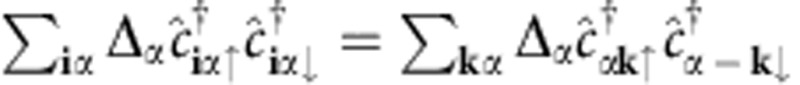. As already discussed, the wavevector **q** enters in the mean-field Hamiltonian in the kinetic energy term through the Peierls substitution (3).

In terms of new fermionic operators 
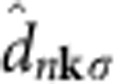
 (see Derivation of the Bogoliubov-de Gennes Hamiltonian in Methods) the mean-field Hamiltonian reads 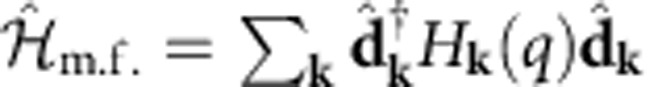 with the Nambu spinor given by 
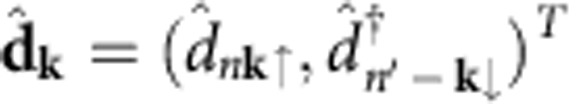
 and the BdG Hamiltonian is a 2 × 2 block matrix defined by


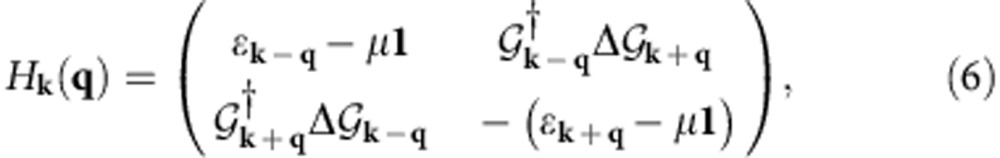


with Δ=Δ*=diag(Δ_*α*_). Due to TRS, the spin index σ has been dropped, and only the eigenvalues and eigenstates for the spin up are used in the following, namely *ɛ*_**k**_=*ɛ*_**k**↑_ and 
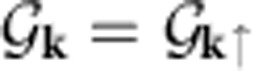
. The BdG Hamiltonian is diagonalized in terms of a diagonal matrix of quasiparticle excitation energies *E*_**k**_(**q**) and a unitary matrix 
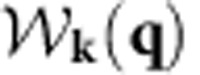



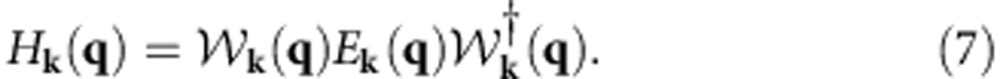


The symmetries of the BdG Hamiltonian for **q**=0 imply that these matrices have the following structure (*E*_*n***k**_>0, see Supplementary Note 3)


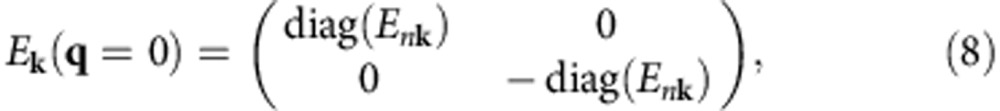



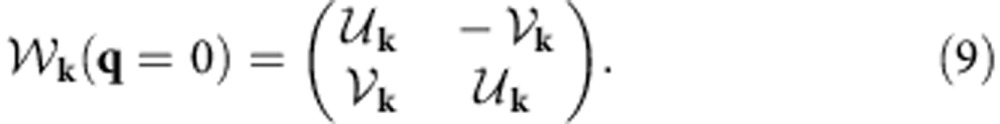


While the kinetic energy terms of the BdG Hamiltonian (6) are diagonal in the band index, the pairing terms depend in a complicated way on the Bloch functions and on the order parameters Δ_*α*_ relative to all orbitals. It is interesting to explore the consequences of this nontrivial structure on superfluid transport. A ‘gauge' transformation of the Bloch functions given by 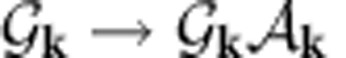, with 
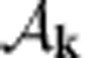
 a unitary matrix subject to the constraint of commuting with the matrix of band dispersions 
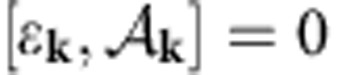
, leaves the BdG Hamiltonian (6) unchanged in form while the eigenfunctions (9) change accordingly. This freedom in the definition of the Bloch function is the same one preventing a unique definition of Wannier functions^24^. All observable quantities, such as current and superfluid weight, are necessarily gauge invariant.

At zero temperature, the grand potential is (see Definition of a generic function of an Hermitian matrix in Methods and Supplementary Note 3)


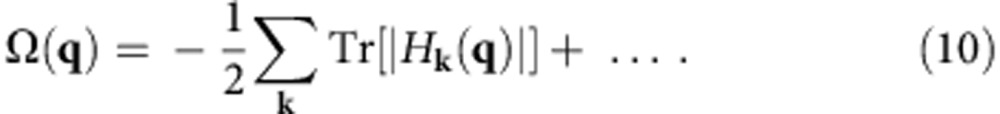


The dots in the above equation represent terms in the grand potential that do not contribute to the superfluid weight. The superfluid current density is obtained from the first derivative of Ω(**q**)


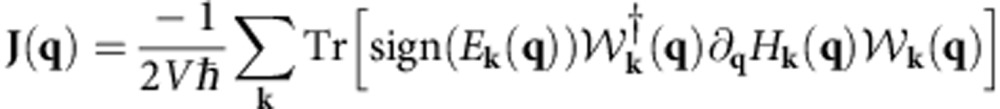



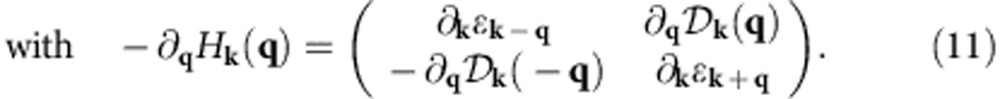


The definition 
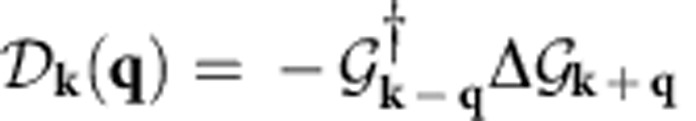
 has been employed above. Due to the linearity of the trace in equation (11), the current splits into two contributions that are separately gauge invariant. We call the first the ‘conventional' current, which depends on the group velocity ∂_**k**_*ɛ*_**k**_/*ħ* and is of order *J*/*ħ*, and another contribution of order Δ/*ħ* that comes from the off-diagonal blocks in equation (11). Our prediction of the latter current component is highly interesting since it may be nonzero in a flat band, unlike the conventional component. Note that in the semiclassical expression for the velocity in a magnetic Bloch band^35^,^36^, two terms appear as well: the group velocity obtained by the band dispersion and the Berry curvature, which is due to interband coupling as the off-diagonal blocks in equation (11). However, the analogy is not complete and the precise relation between Berry curvature and the interband contribution to the superfluid density is clarified below.

The superfluid weight is obtained by taking the derivative of the current density **J**(**q**) and setting **q**=0. The superfluid weight consists of three terms *D*_s_=*D*_s,1_+*D*_s,2_+*D*_s,3_ (details of the derivation are provided in Supplementary Note 3). We call the first term the conventional superfluid weight


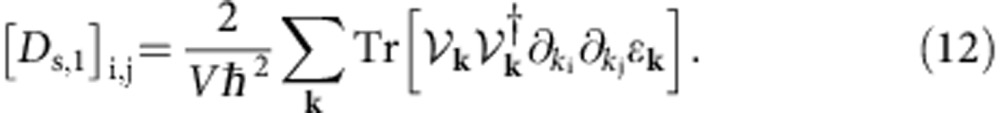


This is the only term present in the single band case, and is zero for a flat band. The other terms are present only in the multiband case. The second term stems from the derivative ∂_*q*_i__ of the off-diagonal blocks in equation (11)


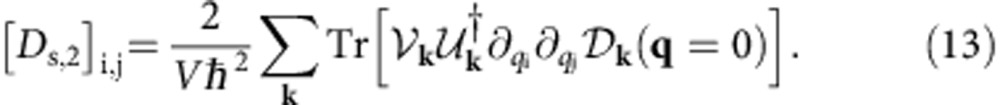


Finally, we have a contribution from terms of the form 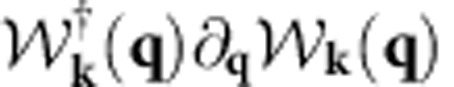:


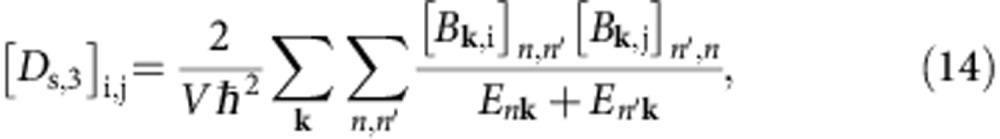



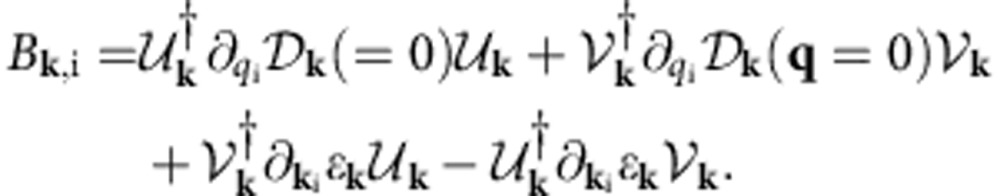


Equations (12)–(14), 39, 41 are the main result of our work since the superfluid weight can be readily calculated using only the ground-state solution (8)-(9). The conventional superfluid weight *D*_s,1_ is invariant under gauge transformations, which means that *D*_s,2_+*D*_s,3_ is itself gauge invariant, thus the superfluid weight splits into two distinct contributions in the same way as the current.

### Superfluid weight in a flat band

The general results in equations (12)–(14), 39, 41 can be specialized to the case of a flat band in two dimensions, and a particularly interesting case is that of a topologically nontrivial flat band. A band specified by 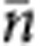 within the composite band 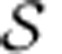 is considered for which the band gaps separating it from the lower 
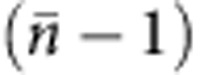
 and upper 
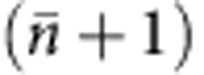
 bands are large with respect to the bandwidth. It is thus possible to have a coupling constant *U* such that


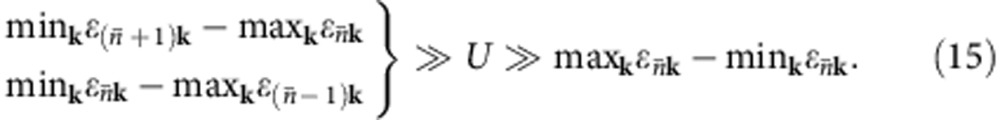


In this limit, the dispersion of the -th band can be approximated by its average 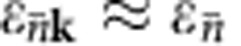. To proceed, it is assumed that the order parameters Δ_*α*_ relative to each orbital in the unit cell are all equal, in other words, that 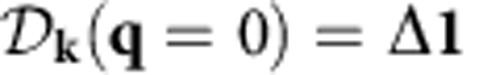 where Δ=Δ_*α*_ is now a real scalar. We can prove this fact rigorously for the Harper–Hubbard model.

Given this assumption, it is shown in Supplementary Note 4 that an approximate self-consistent solution can be found in the limit (15) and has the following form. The matrices 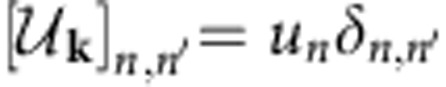 and 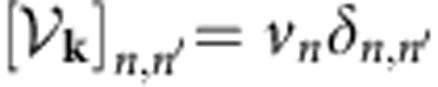 are diagonal, while the other relevant quantities are


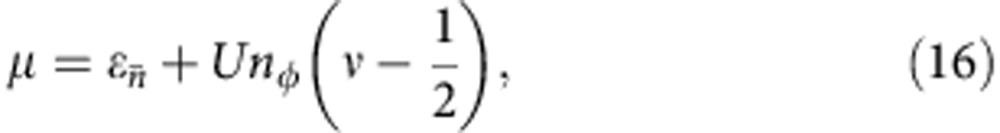



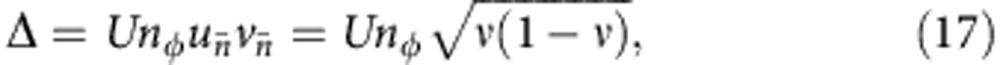



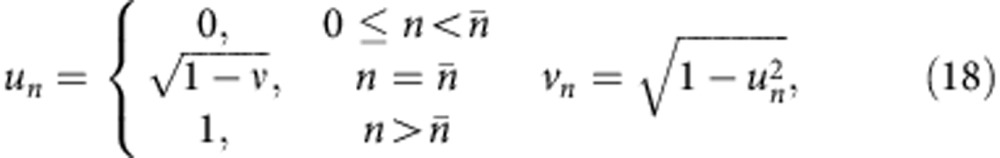



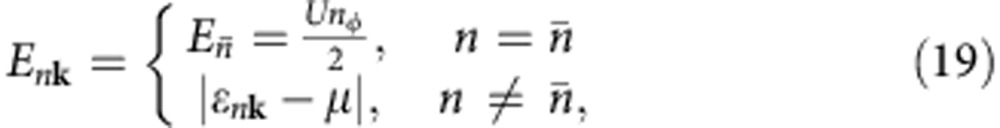


with *ν* the filling factor of the -th band and 
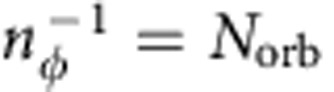
 (the number of orbitals; see Fig. 2a). This solution depends only on equation (15) and is in fact generic for any flat band. The only assumption is Δ_*α*_=Δ.

The above solution can be inserted in the general formulas (12)–(14). The conventional superfluid weight *D*_s,1_ vanishes in the flat band limit, and the remaining part has the form


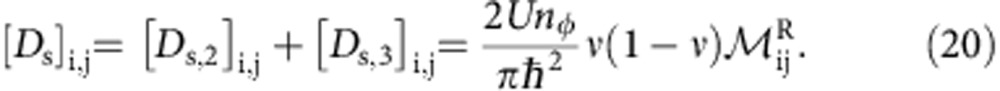


We thus find in the flat-band limit that the superfluid weight is proportional to Δ∝*Un*_*φ*_. This is consistent with ref. 17 for the specific case of the flat band of surface states in rhombohedral graphite, however, our theory is much more general and can be applied to a variety of systems. This result has to be contrasted with the one for an ordinary superconductor in a parabolic band *D*_s_=*n*_p_/*m*_eff_ ∝*J* (with *n*_p_ the total particle density and *m*_eff_ the effective mass) that can be obtained from equation (12), the only term that survives in the single-band case. Therefore, an important prediction is that in a flat-band superconductor, the superfluid weight is linearly dependent on the coupling constant, whereas it is independent from it in an ordinary superconductor. Interestingly, also in superconducting graphene with the chemical potential tuned at the Dirac point, one has^37^,^38^ *D*_s_∝*U*.

The matrix 
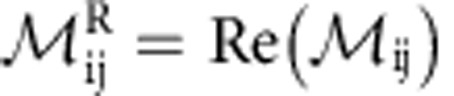
 is the real part of a Hermitian matrix defined as


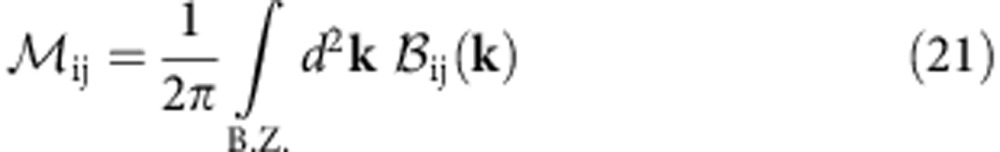


which is the integral over the whole Brillouin-zone of the so-called quantum geometric tensor


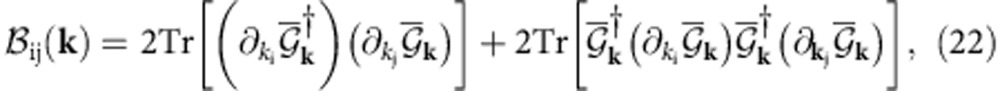


where 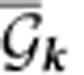 is the projection of 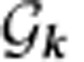 on the -th band (see Positive semidefiniteness of the quantum geometric tensor in Methods). In mathematical terms, the quantum geometric tensor is the Fubini-Study metric in the projective manifold of quantum states^26^,^27^. The quantum geometric tensor has been recently related to observable quantities in a single-particle context such as the noise current spectrum^39^, and plays an important role in characterizing bands that can host fractional Chern insulators, namely, lattice generalization of the fractional quantum Hall state^40^,^41^.

It can be shown that 
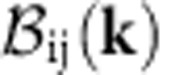
 is zero if 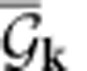 is a square unitary matrix. The case where 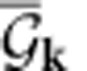 is a square matrix corresponds to superfluid pairing including all the bands of the composite band. Consistently, only if a strict subset of the bands in the composite band participate in the pairing, then the superfluid weight can be nonzero in the flat-band limit. In contrast in the same limit, the whole composite band, which has zero Chern number, is a set of localized orbitals with vanishing hopping. The imaginary part of 
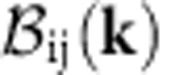
 is the well-known Berry curvature, and its integral over the Brillouin-zone is the Chern number in two dimensions 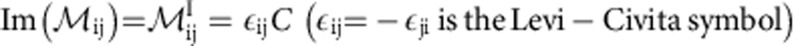. The Chern number refers to spin-resolved bands since the *z* component of the spin is a conserved quantity.

**Figure 1:**
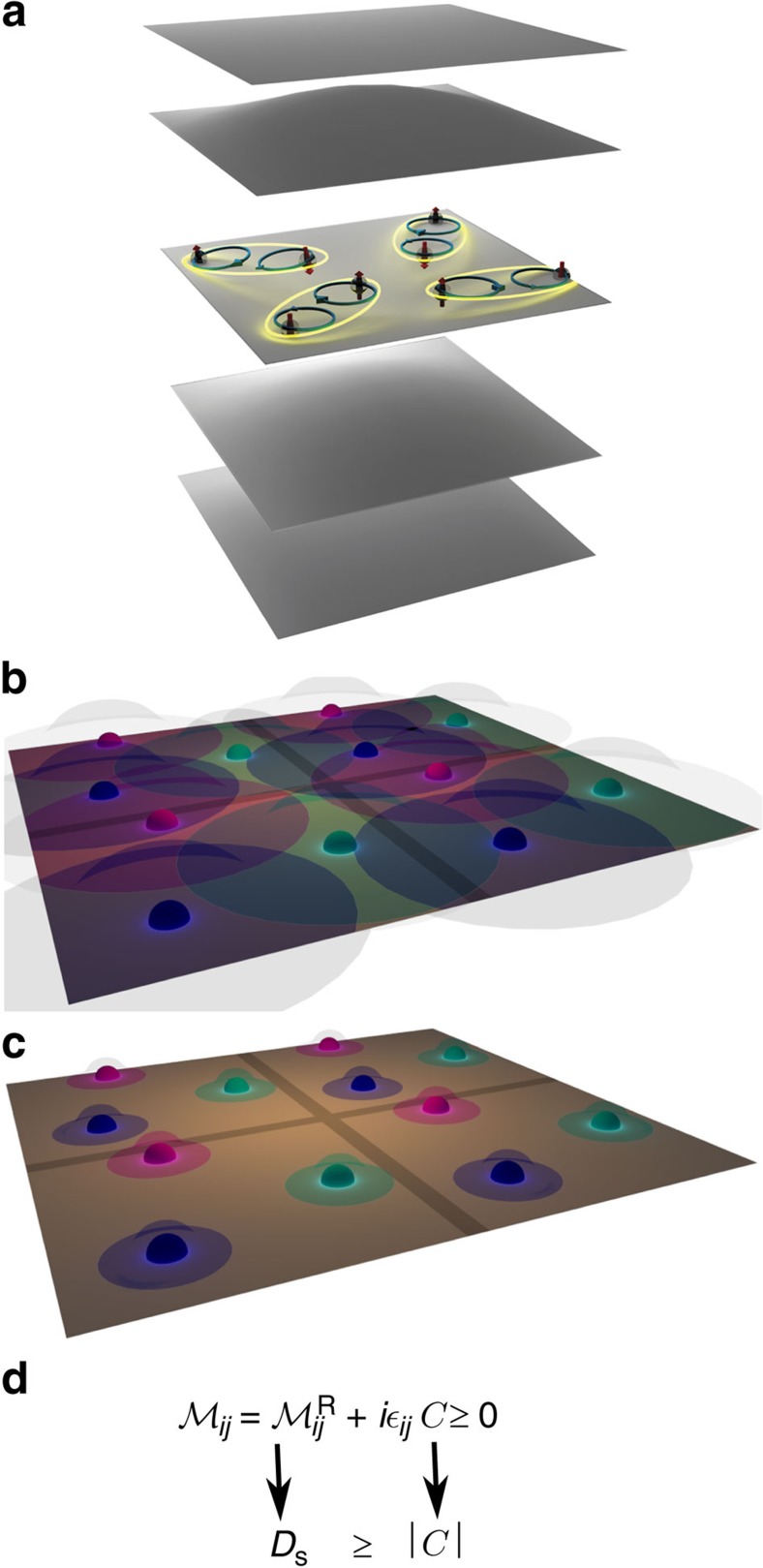
Superfluid transport and Wannier functions. (**a**) Localized Wannier functions are obtained from the Bloch functions of a set of bands, called a composite band^24^,^25^. To have superfluidity in a flat band, the pairing takes place only in a subset of the bands within the composite band, for example, in a single flat band. While the Wannier functions built from the Bloch functions of the band where pairing takes place are delocalized due to the nonzero Chern number^25^ *C*≠0 (**b**) the Wannier functions of the composite band are exponentially localized (**c**). We show that the superfluid weight *D*_s_ in a flat band is given by the Brillouin-zone average of the quantum metric^26^,^27^, which is the real part of an invariant 
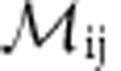
. (**d**) The imaginary part of 
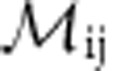
 gives the Chern number *C*. The positivite semidefiniteness of 
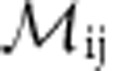
 leads to the bound *D*_s_⩾|*C*| on the superfluid weight.

**Figure 2:**
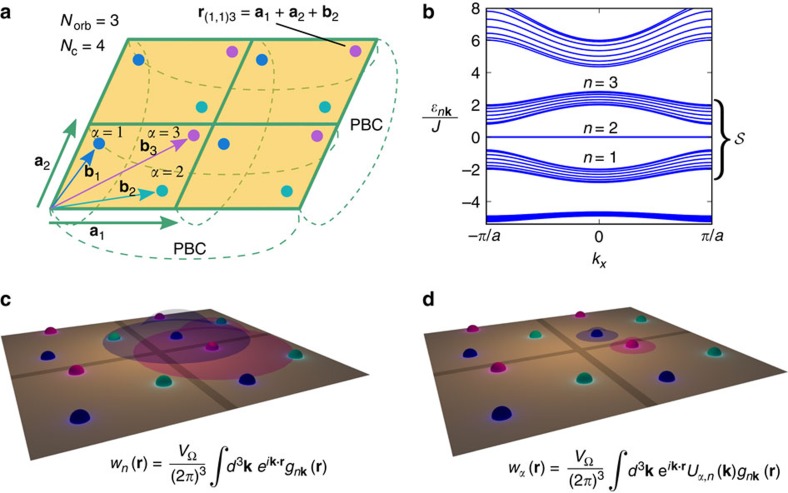
Composite bands and Wannier functions. (**a**) We consider lattices in three and two dimensions (2D in the figure). Periodic boundary conditions (PBC) are used. The lattice contains *N*_c_ unit cells and each unit cell contains *N*_orb_ sites/orbitals. The vectors **a**_*i*_ (i=1, 2, 3) are the fundamental vectors of the Bravais lattice^14^ while the vectors **b**_*α*_ (*α*=1,…,*N*_orb_) are the positions of the centres of the orbitals (Wannier functions) within a unit cell. A single orbital is specified by a triplet (or pair) of integers **i**=(*i*_*x*_, *i*_*y*_, *i*_*z*_) and by the sublattice index *α* and is centred at the position vector **r**_**i***α*_=i_*x*_**a**_1_+i_*y*_**a**_2_+i_*z*_**a**_3_+**b**_*α*_. (**b**) The band structure is obtained by solving the Schrödinger equation 
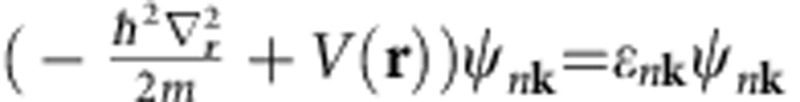
 with periodic potential *V*(**r**)=*V*(**r**+**a**_i_). It consists of the band dispersions *ɛ*_*n***k**_, with *n* the band index and *ħ***k** the lattice quasimomentum, and the periodic Bloch functions *g*_*n***k**_(**r**)=*g*_*n***k**_(**r**+**a**_i_) (Bloch functions for brevity) obtained from the Bloch plane waves 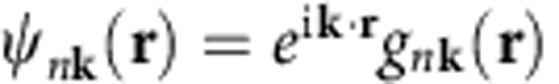. We consider a composite band, that is, a subset 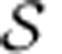 of contiguous bands well separated in energy from other bands. The Chern numbers *C*_*n*_ for individual bands calculated from the Bloch functions may be nonzero (such as the flat band *n*=2 in the figure), but their sum equals zero 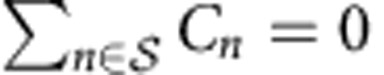. The Chern number refers to spin-resolved bands since the spin along a quantization axis (conventionally the *z* axis) is conserved. (**c**) The Wannier functions, defined as the Fourier transform of the Bloch functions, allow us to derive a tight-binding Hamiltonian that reproduces exactly a single band or a composite band of the original continuum Hamiltonian (see Supplementary Note 2). Since individual bands may be topologically nontrivial with nonzero Chern numbers, their Wannier functions *w*_*n*_(**r**) are not exponentially localized^25^, and the Peierls substitution in the effective Hamiltonian is therefore not justified^15^,^16^. (**d**) By constructing Wannier functions as linear superpositions of Bloch waves of all bands in the composite band, exponentially localized Wannier functions *w*_*α*_(**r**) can be created. The mixing of the different bands is provided by the unitary matrix *U*_*α*,*n*_(**k**). This justifies the Peierls substitution for a composite band 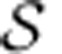.

The real part of 
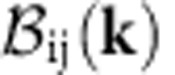
 is a Riemannian metric^26^,^27^ defined over the Brillouin-zone, the so-called quantum metric. In two dimensions, the positive semidefiniteness of the 2 × 2 matrix 
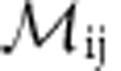
 (see Positive semidefiniteness of the quantum geometric tensor in Methods and Fig. 1) implies that 
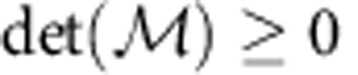
 or


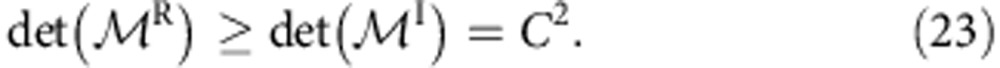


For an isotropic system, the matrix 
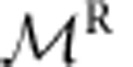
 is proportional to the identity, and this gives the bound *D*_*s*_⩾|*C*|, in appropriate units. The trace Tr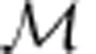 is the gauge invariant part of the localization functional for Wannier functions *F* studied by Marzari and Vanderbilt^39^,^42^, pointing to an intimate connection between non-localization of Wannier functions and the superfluid weight. Indeed, equation (23) also implies that the localization functional is bounded from below by the Chern number. In 2D, the bound is 
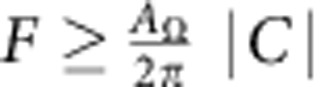
 with *A*_Ω_ the area of the unit cell (see Supplementary Note 5).

### Time-reversal invariant attractive Harper–Hubbard model

To make our results more concrete and study the superfluid weight in a quasi-flat band, we consider the specific example of the time-reversal invariant attractive Harper–Hubbard model^28^. This model is defined on a two dimensional square lattice with lattice spacing *a* by the hopping operator


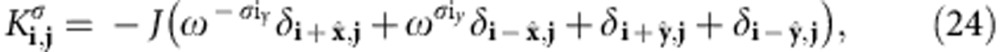


with 
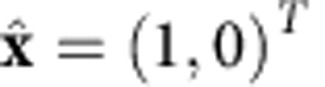
, 
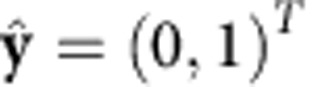
 and 
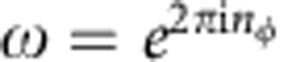
. The phase factors 
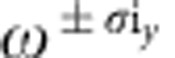
 are the lattice version of the Landau gauge that introduces a uniform magnetic field with flux per plaquette given by *n*_*φ*_. We consider the case of a commensurate flux *n*_*φ*_=1/*Q* with *Q* integer. The magnetic field has opposite signs for opposite spin *σ*=↑(↓)=±. This guarantees that the Hamiltonian is TRS invariant. Since *ω*^*Q*^=1, the discrete translational invariance of the square lattice is broken down to translations by *Q* lattice sites on the *y* direction. We can use the previous notation for composite lattices with the relabelling 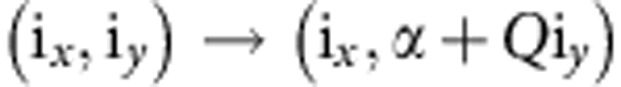. The Bloch functions and band dispersion are solutions of the Harper equation^43^ (Supplementary Note 4).

We are mainly interested in the limit of low flux density per plaquette 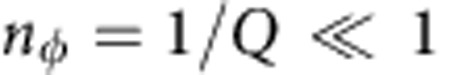. In this case, the bandwidth of each band is exponentially suppressed with respect to the band gap^43^, thus equation (15) is satisfied. As shown in Supplementary Note 4, a self-consistent solution with Δ_*α*_=Δ=const. (Δ is now a real scalar) can be found, and therefore the result for the superfluid weight in equation (20) applies to the Harper–Hubbard model. The only missing piece is the evaluation of 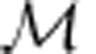 in equations (21) and (22). In the low magnetic field limit, a suitable approximation for the Bloch functions of the lowest bands consistent with the flat-band approximation 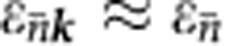 is^43^,


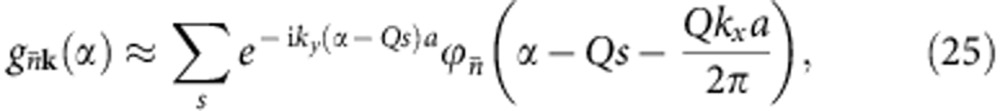


where 
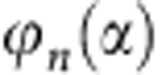
 are the eigenfunctions of the harmonic oscillators if *α* is a continuous variable. In Supplementary Note 4, it is shown that, for the Harper model,


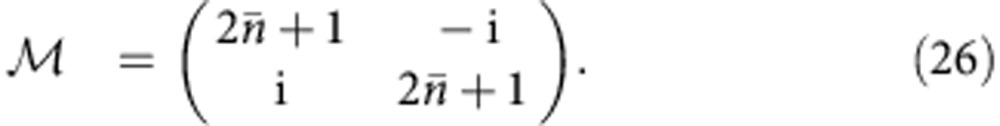


The superfluid weight in the -th band (Landau level in the continuum) is proportional to 
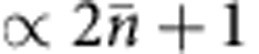
. Note how the bound (23) is saturated for the lowest Landau level (all Landau levels in the continuum have^20^,^43^ |*C*|=1). By working directly in the continuum, we have obtained precisely the result contained in equations (20) and (26) (details are not provided here). More generally, Equations (20)–(22), 61, 62 are valid for a generic flat band, while equation (26) is specific for the Harper Hamiltonian.

## Discussion

We have discovered that an invariant built from the quantum geometric tensor, which is intimately related to the Chern number, governs superfluidity in the flat-band limit. The inequality (23) implies that a topologically nontrivial flat band (*C*≠0) is guaranteed to have a finite superfluid density in the presence of pairing in the system. Similar but more complicated bounds are also expected in three dimensions, since 
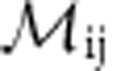
 is positive semidefinite in general, and its imaginary part encodes three Chern numbers instead of one. This is the first time that the superfluid weight has been directly related to a topological invariant. Remarkably, BdG Hamiltonians with TRS and invariance under spin rotation around a given axis belong to the chiral unitary class AIII, whose ground state is topologically trivial in 2D according to the classification of ref. 18, therefore, we are referring to bulk superfluid transport and not to transport due to edge modes.

In a flat band, mean-field theory is usually not adequate, however, the BCS wavefunction, implicit in the BdG approach, is the exact ground state in the continuum limit of the Harper–Hubbard model considered here. This can be shown by mapping to the wavefunction of a quantum Hall ferromagnet^44^,^45^,^46^ (see Exactness of the BCS wavefunction in Methods). Under this mapping, the result given by (20) and (26) for the superfluid weight of the Harper–Hubbard model translates into the spin stiffness or, equivalently, the counterflow-current superfluid density of a quantum Hall ferromagnet^44^,^46^ with contact repulsive interactions. Whether mean-field theory can describe pairing in flat bands other than Landau levels is an open problem, analogous to the problem of characterizing the bands that can host a fractional Chern insulator^40^,^41^, but considerably less studied. We have checked that dynamical mean-field theory calculations (which treat local fluctuations exactly) for the Harper–Hubbard model are indeed in excellent agreement with mean-field theory in the case of quasi-flat bands^47^.

Another problem of mean-field theory in 2D is that the transition to the normal state occurs at the Berezinsky–Kosterlitz–Thouless (BKT) transition temperature *T*_BKT_, which is related to the superfluid density by a universal relation and is lower than the mean-field critical temperature. At half filling, the estimated *T*_BKT_ is close to the mean-field transition temperature *T*_c_ (see Supplementary Note 6 and Supplementary Fig. 1)


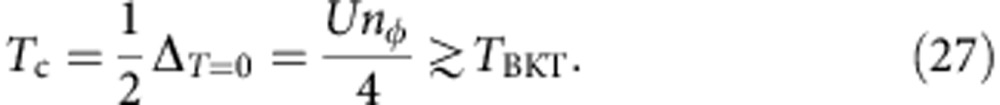


Indeed, we find *T*_BKT_≈0.25, 0.61, 0.75*T*_c_ for 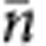
=0, 1, 2, respectively.

The superfluid weight is a linear response transport coefficient, a ground-state property, and it can be calculated exactly if the exact ground state is known^46^, as in the case of the Harper–Hubbard model discussed above. As a consequence, it is not is necessary to employ beyond mean-field methods for estimating the superfluid weight^48^. In summary, while the validity of mean-field theory for flat bands is in general an open question, the superfluid weight derived here for the Harper–Hubbard model is exact in the flat-band limit and a good approximation for quasi-flat bands.

In ultracold gases, the atom–atom interaction is tunable, thus these systems are an ideal platform to confirm our prediction that in a flat-band *D*_s_∝*U*. In fact, it is possible to introduce complex hoppings in a lattice Hamiltonian (Peierls substitution) by Raman dressing^49^ or lattice shaking^50^. Notably, the Harper model has been recently implemented with ultracold gases^29^,^30^. Whereas at the qualitative level superfluidity in ultracold gases is a well-established fact, a quantitative measurement of the superfluid weight has not been easy to perform so far. It has been proposed that the superfluid weight can be measured by an analogue of the classic Adronikashvili experiment^51^, whereas the superfluid fraction of the unitary Fermi gas has been measured by means of second sound^52^. Moreover, recent transport experiments with ultracold Fermi gases^53^,^54^ make it realistic to measure quantities like the superfluid weight. Currently, the main issue in ultracold gas experiments is the excessive heating present in experiments with artificial gauge fields^30^. Our estimates indicate (see Estimate of the critical temperature for the Harper–Hubbard model in Methods) that superfluidity may be achieved in the future in topologically nontrivial flat bands that can be realized with ultracold atoms. Flat bands have been suggested as a possible mechanism to explain high-*T*_c_ superconductors^11^,^10^, and our results can be used to prove this hypothesis. If our results are generalized to the long-range Coulomb interaction, then one more experimental context where they may be important are quantum Hall ferromagnets (cf. the above-mentioned mapping of the superfluid weight (equations (20) and (26)) to the spin stiffness of a quantum Hall ferromagnet). In fact, a contact interaction is not an acceptable approximation in this case.

Our results can be understood by distinguishing two possible ways to obtain a band of exactly degenerate states. On one hand, the particles can be confined in states with negligible overlap by high potential barriers, or alternatively localization can occur in overlapping orbits due to (pseudo-)magnetic fields or lattice geometry. In the latter case, the possibility of transport is a nontrivial question. The fact that we find a nonzero superfluid weight in a flat band can be understood by finite overlap of the Cooper pairs, indeed pairing fluctuations support transport whenever Cooper pairs can be created and destroyed at distinct locations. Somewhat related in a work^55^ that focused on condensation rather than superfluidity, an effective Hamiltonian for bosons in a flat band was derived by taking matrix elements of the interaction between overlapping Wannier functions, which produced an effective hopping for the particles. In the work of Provost and Vallee^26^, pointing out for the first time the natural geometric structure present in a manifold of quantum states, it is suggested that macroscopic quantum systems that exhibit collective behaviour might be those where the quantum metric has direct physical significance, an intuition that has, in some sense, materialized in our results that showed the connection between quantum metric and superfluid weight. It is an intriguing topic for future research to understand whether the pairing fluctuations and macroscopic phase of a superfluid have any connection to the fact that the quantum metric equals the fluctuations in the quantity that generates the path of a quantum state in the manifold^26^.

While the above discussion may help to guide the intuition, the rigorous framework for future work is given by our results on the important role of Wannier functions in superfluid transport. As we have shown, the bound on the superfluid weight translates into a bound on the the localization functional for Wannier functions^42^. A nonzero Chern number implies that the Wannier functions have algebraically decaying tails^24^, and this explains the bound *D*_s_⩾|*C*|. But the Wannier functions can also be delocalized on a short range only, which is consistent with the fact that the superfluid weight is related to an invariant distinct from the Chern number. In general, we propose (quasi-)flat bands as a viable way to increase the critical temperature in novel superconducting materials, while at the same time preserving the defining properties of superconductors. We expect the invariant 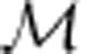 that controls the superfluid weight in a flat band to play a central role in this research effort.

## Methods

### Derivation of the Bogoliubov-de Gennes Hamiltonian

The BdG Hamiltonian in equation (6) is important for our purposes, and here we clarify its derivation. The hopping matrix has the same discrete translational symmetry as the Bravais lattice, since 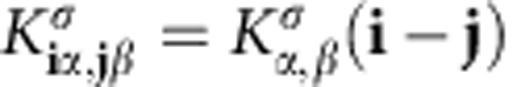. By expanding the field operators into plane waves 
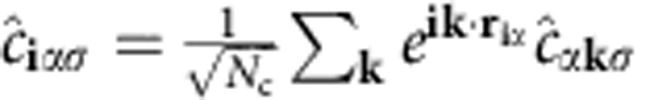
, the kinetic term of the Hamiltonian can be block-diagonalized in momentum space


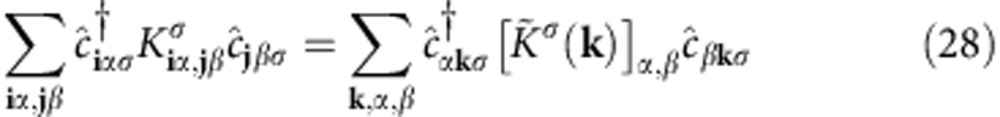



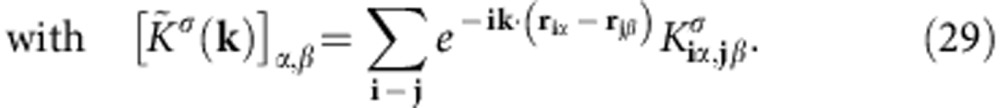


It is convenient to introduce a Nambu spinor 
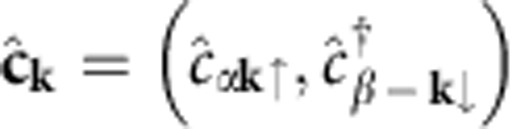
 built out of the operators 
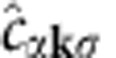
 in the plane wave basis (see equation (28)) and write the mean-field Hamiltonian as 
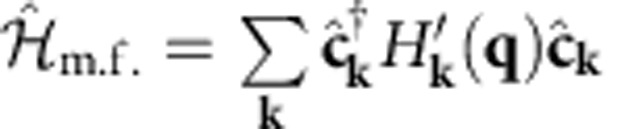
 with


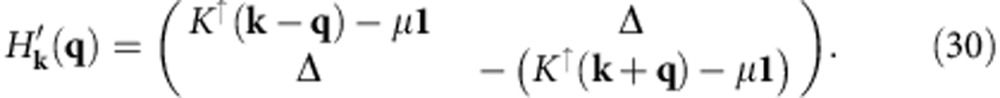


To cast the mean-field Hamiltonian in Nambu form, we have anticommuted the spin-down creation and annihilation operators in the kinetic energy term and used TRS in the form (*K*^↓^(**k**))*=*K*^↑^(−**k**). All the c-number terms in the mean-field Hamiltonian have been dropped since they do not affect the superfluid weight (see Supplementary Notes 1 and 3). A further canonical transformation is performed to go from the basis given by the orbitals within a unit cell, labelled by *α*,*β*, to the basis that diagonalizes the kinetic Hamiltonian, that is, the Bloch functions labelled by *n*. More precisely, the transformation reads 
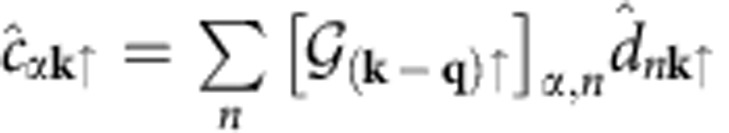
 and 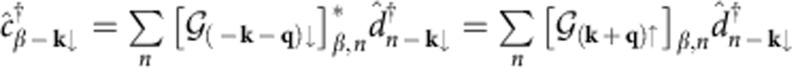. In this way, equation (6) is obtained.

### Definition of a generic function of an Hermitian matrix

In equations (10) and (11), the absolute value |·| and the sign function sign(·) of the BdG Hamiltonian *H*_**k**_(**q**) are used. In general, a function *f*(·) of an Hermitian matrix *H*=*UDU*^†^, diagonalized by the unitary matrix *U* and by the real diagonal matrix *D*, is defined as the function of the eigenvalues *f*(*H*)=*Uf*(*D*)*U*^†^.

### Positive semidefiniteness of the quantum geometric tensor

In equation (22), the projection 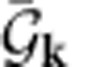 of the unitary matrix 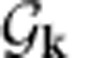 on the -th band is defined by


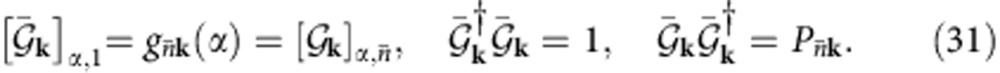



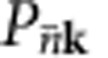
 is a projection operator, a positive semidefinite 
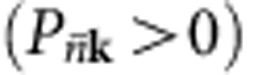
 and idempotent 
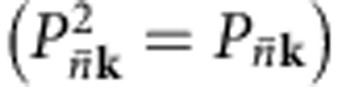
 operator. The matrix 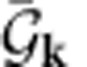 is just a column vector in equation (31), but it can be a rectangular matrix for a group of degenerate flat bands, for example. Since the dispersion is flat, 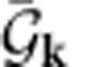 characterizes the flat band completely. The positive semidefiniteness of the projector *P*_*n***k**_ and of its complement **1**−*P*_*n***k**_ implies that the matrix 
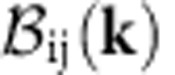
 in equation (22) is positive semidefinite since it can be written in the form


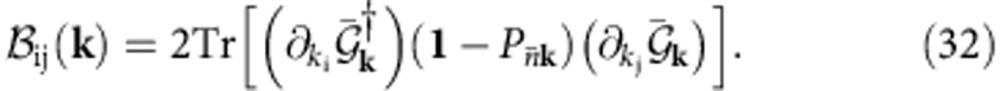


The invariant matrix in equation (21) is also positive semidefinite 
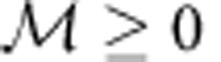
 since it is a linear combination with positive coefficients of the positive semidefinite matrices 
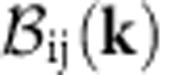
. Interestingly, the Berry curvature (
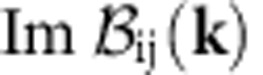
) and the Chern number of a set of bands are obtained by adding the respective contributions of all bands in the set, whereas the quantum metric 
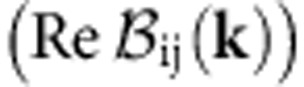
 is not additive due to the second term in equation (22), which is real and involves a double sum over the band index.

### Exactness of the BCS wavefunction

The BCS wavefunction can be shown to be the exact ground state of the Harper–Hubbard model in the flat-band limit. To take the flat-band limit of the Harper–Hubbard model, it is necessary to take the limit of low magnetic flux. The problem is mapped into that of particles in the continuum in the presence of a constant magnetic flux (Landau problem). We consider a general form for the interparticle interaction potential *V*(**r**)=(2*π*)^−2^∫*d*^2^**q***v*(**q**)*e*^i**q**·**r**^ and perform the projection of the interaction term into the -th Landau level^41^


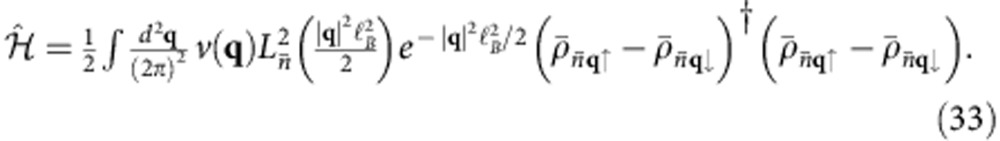


Here 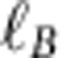 is the magnetic length, *L*_*n*_(*x*) is the *n*-th Laguerre polynomial and 
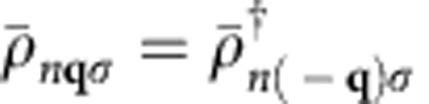
 are projected density operators that obey the Girvin–MacDonald–Platzman algebra


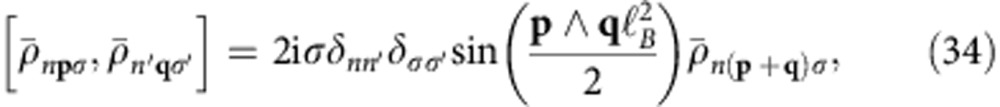


where 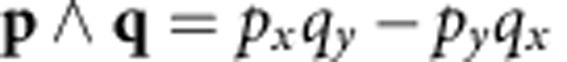. In the Landau gauge, the explicit expression for the projected density operators is


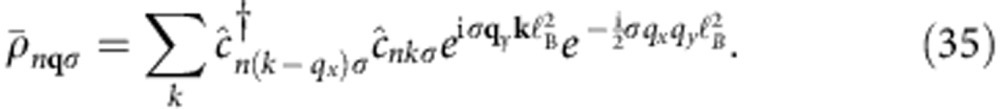


The annihilation (creation) operators 
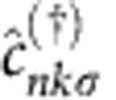
 are labelled by the Landau level index *n* and the momentum *k* along the *x* direction which is conserved in the Landau gauge. Notice that if *v*(**q**)⩾0, the interaction Hamiltonian (33) is repulsive between particles with the parallel spins and attractive between particles with antiparallel spins. It is straightforward to verify that the operator 
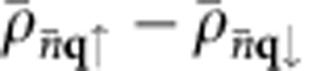
 in the Hamiltonian annihilates the BCS wavefunction


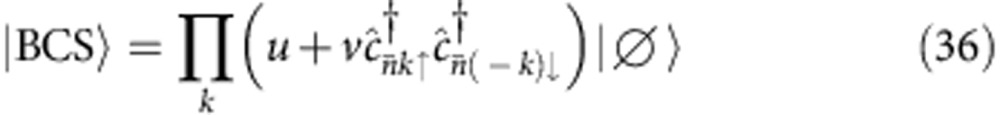


for arbitrary values of *u* and *v* and any value of **q**, that is, the BCS wavefunction is a zero eigenvector of the Hamiltonian. Normalization requires that |*u*|^2^+|*v*|^2^=1. A possible parametrization is 
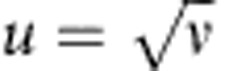
 and 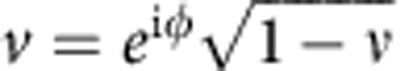 with *ν* the filling and *e*^i*φ*^ an arbitrary phase. Since the Hamiltonian (33) is a positive semidefinite operator for *v*(**q**)⩾0, the BCS wavefunction must be the ground state since it is a zero eigenvector.

An alternative way to interpret this result is well known in the context of quantum Hall physics^44^,^46^. By performing a particle-hole transformation of the form 
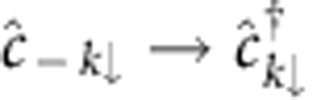
, the BCS wavefunction is transformed into the wavefunction of a completely polarized ferromagnet


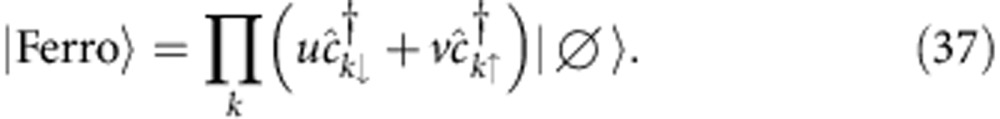


This is a simple Slater determinant where all the states with spin wavefunction 
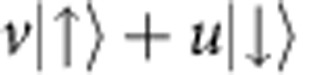
 are occupied. Under the same transformation, the interparticle interaction becomes a repulsive interaction, which is completely isotropic in spin space. It is easy to understand why the wavefunction (Equation (37)) is the ground state. According to Hund's rule, the interaction energy is minimized if the all the spins are parallel (a consequence of the Pauli exclusion principle), and in a Landau level, there is no kinetic energy cost that prevents a complete alignment. Indeed, this extreme ferromagnetic state has been observed in experiments in the quantum Hall regime^44^. It is important to note that the *z* component of the magnetization in the ferromagnetic state is mapped by the particle-hole transformation into the total number of particles on the superconducting side (and vice versa). Therefore, whereas the wavefunction (37) is the ground state when a spinful Landau level is half-filled, the BCS wavefunction is the correct ground state for any filling.

In the limit of a contact interaction, the repulsive interaction between particles with parallel spins disappears and one is left with a purely attractive interaction, that is, the continuum limit of the Harper–Hubbard model considered here.

### Estimate of the critical temperature for the Harper–Hubbard model

To estimate the critical temperature for an actual ultracold gas experiment, we consider fermionic ^6^Li atoms in an optical lattice with a typical wavelength of the laser standing wave *λ*=1,064 nm=2*π*/*k* and the corresponding recoil energy given by 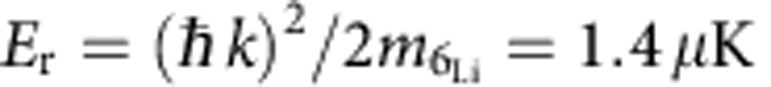. The hopping energy scale *J* can then be estimated from the approximate formula


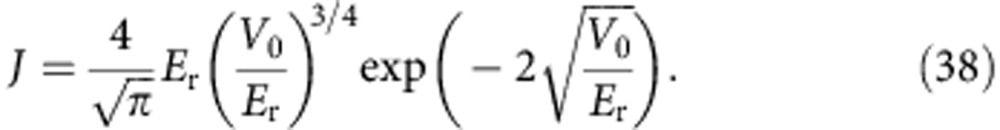


Using the same ratio *V*_0_/*E*_r_≈7 as in ref. 29 between the amplitude *V*_0_ of the optical lattice potential and the recoil energy, one obtains *J*≈70 nK. In Supplementary Note 4 and Supplementary Fig. 2, we estimate that in the isolated flat-band approximation for the time-reversal invariant attractive Harper–Hubbard model, the mean-field critical temperature is of the order of *k*_B_*T*_c_≈0.02*J*, which implies a BKT transition temperature in the order of the nanoKelvin. Such a low temperature results just because we wished to be able to use the analytical results derived here, which requires pairing within a single band and thus *U* needs to be smaller than the gaps to neighbouring bands, equation (15). Conceptually the same results can, however, be achieved when several (but not all) flat (or nearly flat) bands of the composite bands participate in pairing, only that the theoretical analysis becomes more involved. Then, the limit on *U* is relaxed, and *T*_c_ can be substantially increased.

## Additional information

**How to cite this article:** Peotta, S. & Törmä, P. Superfluidity in topologically nontrivial flat bands. *Nat. Commun.* 6:8944 doi: 10.1038/ncomms9944 (2015).

## Supplementary Material

Supplementary Information Supplementary Figures 1-2, Supplementary Notes 1-6 and Supplementary References
