# Supplementary material for: Superfluidity in topologically nontrivial flat bands
Source: Nat Commun. 2015 Nov 20;6:8944. doi: 10.1038/ncomms9944 (PMC4673883; doi:10.1038/ncomms9944)
Supplement: Supplementary Information — Supplementary Figures 1-2, Supplementary Notes 1-6 and Supplementary References [file ncomms9944-s1.pdf]

Supplementary Figures

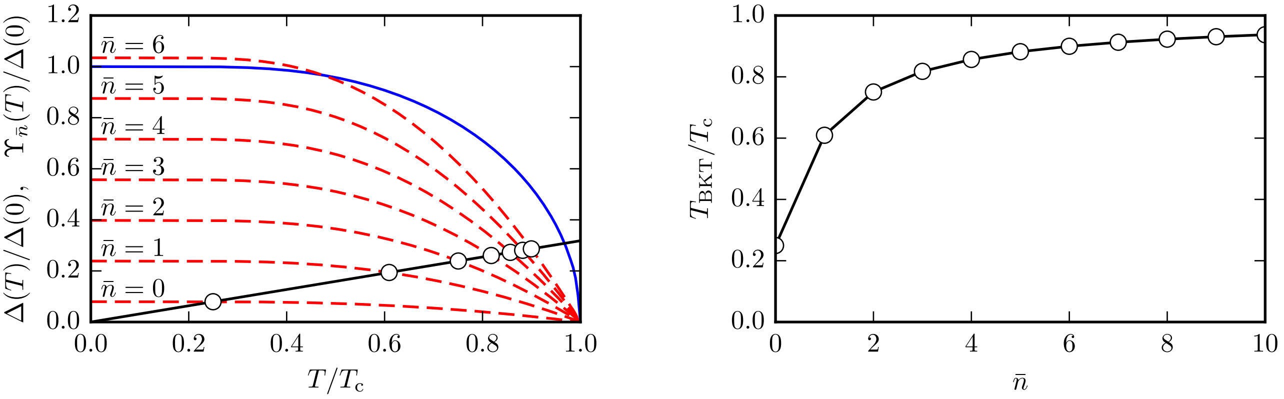

Supplementary Figure 1: On the left plot the energy gap $\Delta(T)/\Delta(0)$ (blue solid line) and the stiffness $\Upsilon_{\bar{n}}(T)/\Delta(0)$ (red dashed line) as a function of temperature are show. The stiffness $\Upsilon_{\bar{n}}$ is shown for several values of $\bar{n}$. The intercepts between the black straight line and the red dashed lines correspond to the solutions of Eq. (95) which gives the BKT temperature $T_{\rm BKT}$ for several values of $\bar{n}$. The calculated solutions of Eq. (95) are shown in the right plot. Only half-filled flat bands ($\nu = 1/2$) are considered here.

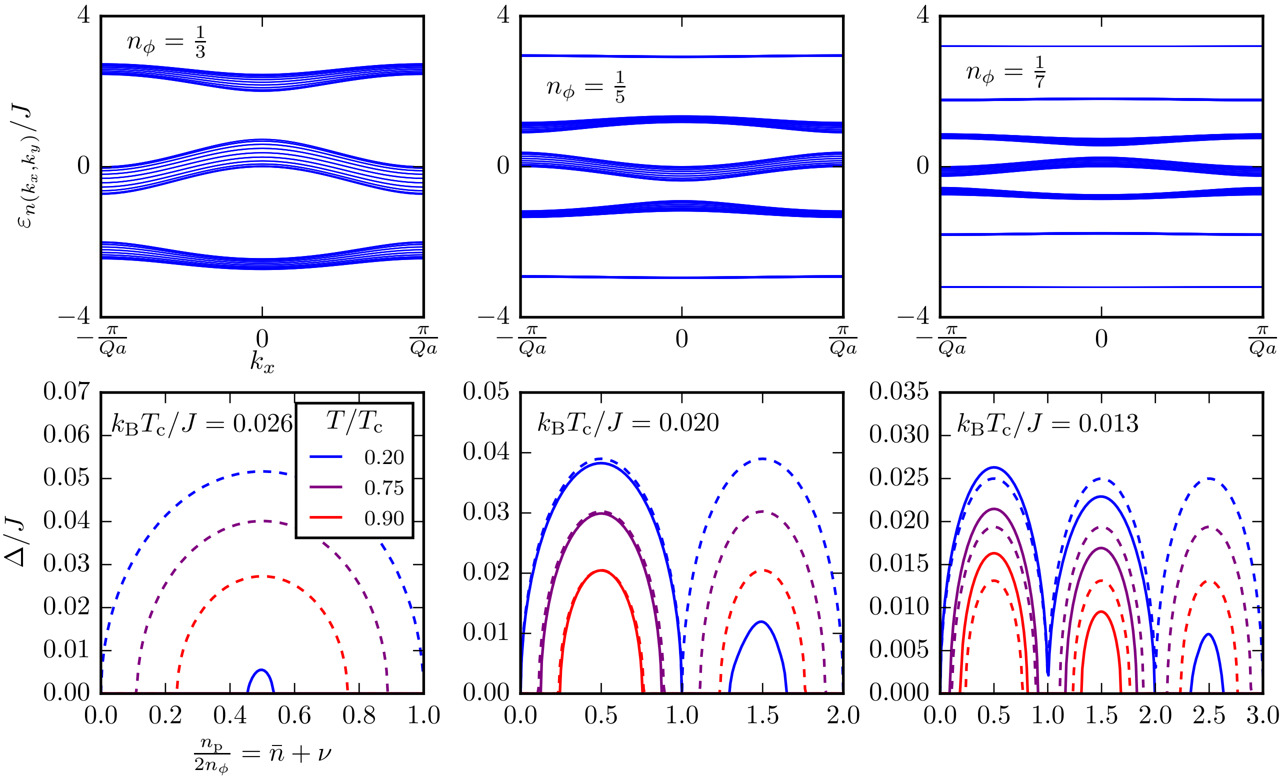

Supplementary Figure 2: In the upper panels the band structure of the Harper model for three different values of the number of magnetic flux quanta per plaquette $n_\phi = 1/Q = \frac{1}{3}, \frac{1}{5}, \frac{1}{7}$ is shown. The band dispersion $\varepsilon_{n\mathbf{k}}$ is shown as a function of the wavevector k_x for twenty different values of k_y. Both k_x, k_y are restricted to the reduced Brillouin zone $k_x, k_y \in [-\pi/(Qa), \pi/(Qa)]$ due to the degeneracy of the dispersion in the Harper model (53). Notice how for decreasing n_ϕ the lower bands approach the flat-band limit. In the lower panel the corresponding energy gap Δ calculated self-consistently by using Eq. (59)-(60) (solid lines) is shown as a function of the normalized total particle density $n_\mathrm{p}/(2n_\phi) = \bar{n} + \nu$. The value of the coupling constant U/J in all three cases is approximately equal to one-fourth the energy gap between the two lowest bands, more precisely $U/J = 0.31$ for $n_\phi = 1/3$, $U/J = 0.39$ for $n_\phi = 1/5$ and $U/J = 0.35$ for $n_\phi = 1/7$. The critical temperature $k_\mathrm{B}T_\mathrm{c} = \frac{Un_\phi}{4} = \Delta_{T=0}/2$ shown in the lower panels is the mean-field critical temperature in the isolated flat-band limit. The numerical self-consistent solution of Eq. (59)-(60) is compared to the approximate solution in the isolated flat-band limit (Eq. (61)-(64)) (dashed lines) for three different temperatures $T/T_\mathrm{c} = 0.2, 0.75, 0.9$. The isolated flat-band approximation is not good for any band in the case $n_\phi = 1/3$, but it can be used for the first band for $n_\phi = 1/5$ and for the first two bands for $n_\phi = 1/7$.

Supplementary Note 1: Superfluid weight from the grand potential

The aim of this Supplementary Note is to justify Eq. (1) in the main text stating that the superfluid weight can be calculated by taking successive derivatives of the grand potential $\Omega(T, \Delta, \mu, \mathbf{q})$ only with respect to the wavevector $\mathbf{q}$. The gap function $\Delta(\mathbf{q})$ and the chemical potential $\mu(\mathbf{q})$ are themselves a function of $\mathbf{q}$ (for fixed particle density $n_{\rm p}$). This dependence is in principle important and requires the solution of the self-consistency equations in Δ and μ for nonzero values of $\mathbf{q}$. It is shown here that this not the case. In the following we do not make use of translational invariance since the result that we want to prove holds in general. The greek indices α, β that appear in the case of a composite lattice will be suppressed as well since what we are going to prove holds equally well in single-band and multiband systems. The mean-field decoupling of the Hubbard interaction is (including the c-number term)

$$-U \sum_{\mathbf{j}} \hat{c}^\dagger_{\mathbf{j}\uparrow}\hat{c}^\dagger_{\mathbf{j}\downarrow}\hat{c}_{\mathbf{j}\downarrow}\hat{c}_{\mathbf{j}\uparrow} \approx \sum_{\mathbf{j}} \left[\Delta_{\mathbf{j}}\hat{c}^\dagger_{\mathbf{j}\uparrow}\hat{c}^\dagger_{\mathbf{j}\downarrow} + \Delta^*_{\mathbf{j}}\hat{c}_{\mathbf{j}\downarrow}\hat{c}_{\mathbf{j}\uparrow} + \frac{|\Delta_{\mathbf{j}}|^2}{U}\right] . \tag{1}$$

It will be shown below that the self-consistency equation for the pairing order parameter (also called energy gap function) $\Delta_{\mathbf{j}} = -U\langle \hat{c}_{\mathbf{j}\downarrow}\hat{c}_{\mathbf{j}\uparrow}\rangle$ can be obtained as an extremizing condition for the grand potential.

The mean-field Hamiltonian $\hat{\mathcal{H}}_{\rm m.f.}$, comprising the single particle (quadratic) term in Eq. (3) in the main text and the mean field approximation (1) for the interaction term can be conveniently rewritten in matrix form in Nambu space

$$\hat{\mathcal{H}}_{\rm m.f.} = \hat{\mathbf{c}}^\dagger H_{\rm BdG}(\mathbf{q})\hat{\mathbf{c}} + \mathrm{Tr}\, H^{\downarrow}(\mathbf{q}) + \frac{1}{U}\sum_{\mathbf{i}} |\Delta_{\mathbf{i}}(\mathbf{q})|^2 , \tag{2}$$

where the following definitions have been used

$$\hat{\mathbf{c}} = \begin{pmatrix} \hat{c}_{\mathbf{j},\uparrow} \\ \hat{c}^\dagger_{\mathbf{j},\downarrow} \end{pmatrix} \quad \text{(Nambu spinor)} , \tag{3}$$

$$H_{\rm BdG}(\mathbf{q}) = \begin{pmatrix} H^{\uparrow}_{\mathbf{i},\mathbf{j}}(\mathbf{q}) & \Delta_{\mathbf{i}}(\mathbf{q})\delta_{\mathbf{i},\mathbf{j}} \\ \Delta^*_{\mathbf{i}}(\mathbf{q})\delta_{\mathbf{i},\mathbf{j}} & -H^{\downarrow}_{\mathbf{i},\mathbf{j}}(\mathbf{q}) \end{pmatrix} . \tag{4}$$

$$H^{\uparrow}_{\mathbf{i},\mathbf{j}}(\mathbf{q}) = K^{\uparrow}_{\mathbf{i},\mathbf{j}}(\mathbf{q}) - \mu(\mathbf{q})\delta_{\mathbf{i},\mathbf{j}} + V_{\mathbf{i}}\delta_{\mathbf{i},\mathbf{j}} , \tag{5}$$

$$H^{\downarrow}_{\mathbf{i},\mathbf{j}}(\mathbf{q}) = \left(K^{\downarrow}_{\mathbf{i},\mathbf{j}}(\mathbf{q})\right)^* - \mu(\mathbf{q})\delta_{\mathbf{i},\mathbf{j}} + V_{\mathbf{i}}\delta_{\mathbf{i},\mathbf{j}} = K^{\uparrow}_{\mathbf{i},\mathbf{j}}(-\mathbf{q}) - \mu(\mathbf{q})\delta_{\mathbf{i},\mathbf{j}} + V_{\mathbf{i}}\delta_{\mathbf{i},\mathbf{j}} , \tag{6}$$

The trace in Eq. (2) comes from the constant term produced when anticommuting fermion operators $\hat{c}^\dagger_{\mathbf{i}\sigma}\hat{c}_{\mathbf{j}\sigma} = \delta_{\mathbf{i},\mathbf{j}} - \hat{c}_{\mathbf{j}\sigma}\hat{c}^\dagger_{\mathbf{i}\sigma}$ ($\delta_{\mathbf{i},\mathbf{j}}$ is the Kronecker delta function). In our case we have anticommuted the fermionic operators relative to the down spin.

In the above equations several quantities depend on the wavevector $\mathbf{q}$. The hopping matrix $K^{\sigma}_{\mathbf{i},\mathbf{j}}(\mathbf{q}) = K_{\mathbf{i},\mathbf{j}}e^{i\mathbf{q}\cdot(\mathbf{r}_{\mathbf{i}}-\mathbf{r}_{\mathbf{j}})}$ depends on $\mathbf{q}$ according to the Peierls substitution, Eq. (2) in the main text. The pairing potential $\Delta_{\mathbf{i}}(\mathbf{q})$ is a function of $\mathbf{q}$ due to the fact that the mean-field Hamiltonian (2) must be solved self-consistently, while the chemical potential depends on $\mathbf{q}$ since the particle number per lattice site $n_{\rm p}$ is fixed. We have introduced an additional spin-independent scalar potential $V_{\mathbf{i}}$ in the diagonal terms of $H_{\rm BdG}$ (see Eq. (5)-(6)) since our derivation applies to a system without translational invariance. In Eq. (6) we have used the time-reversal symmetry (TRS) of the hopping term $\left(K^{\downarrow}_{\mathbf{i},\mathbf{j}}(\mathbf{q})\right)^* = K^{\uparrow}_{\mathbf{i},\mathbf{j}}(-\mathbf{q})$. Notice that a finite wavevector $\mathbf{q}$ breaks TRS since it has the effect of inducing a finite supercurrent in the ground state. However TRS connects the Hamiltonians with opposite wavectors $\mathbf{q} \to -\mathbf{q}$, in fact under TRS the sign of the supercurrent is reversed.

A basic thermodynamic function we are interested in is the thermodynamic grand potential Ω defined from the grand partition function $\mathcal{Z}_\Omega$

$$\Omega\big(T, \mu(\mathbf{q}), \Delta_{\mathbf{i}}(\mathbf{q}), \mathbf{q}\big) = -\frac{1}{\beta}\ln \mathcal{Z}_\Omega = -\frac{1}{\beta}\ln \mathrm{Tr}[e^{-\beta\hat{\mathcal{H}}_{\rm m.f.}}] = \mathrm{Tr}\, H_{\downarrow}(\mathbf{q}) + \sum_{\mathbf{i}}\frac{|\Delta_{\mathbf{i}}(\mathbf{q})|^2}{U} - \frac{1}{\beta}\ln \mathrm{Tr}[e^{-\beta\hat{\mathbf{c}}^\dagger H_{\rm BdG}(\mathbf{q})\hat{\mathbf{c}}}] . \tag{7}$$

The grand potential is a function of the wavevector $\mathbf{q}$ either directly, since $\mathbf{q}$ appears in the diagonal blocks of $H_{\text{BdG}}(\mathbf{q})$ through the kinetic energy term $K^\sigma(\mathbf{q})$ [see Eq. (4)] or indirectly through the quantities $\mu(\mathbf{q}), \Delta_{\mathbf{i}}(\mathbf{q})$. This distinction is important for what follows. The last term on the right hand side of the above equation can be evaluated by diagonalizing the BdG Hamiltonian. We use the notation

$$H_{\text{BdG}}(\mathbf{q}) = \mathcal{W}(\mathbf{q}) E(\mathbf{q}) \mathcal{W}^\dagger(\mathbf{q})\,. \tag{8}$$

The matrix $\mathcal{W}(\mathbf{q})$ is unitary, while $E(\mathbf{q})$ is diagonal and the diagonal elements are the eigenvalues $E_a(\mathbf{q})$, namely

$$[E(\mathbf{q})]_{a,b} = E_a(\mathbf{q})\delta_{a,b} = E_a\big(\mu(\mathbf{q}), \Delta_{\mathbf{i}}(\mathbf{q}), \mathbf{q}\big)\delta_{a,b}\,. \tag{9}$$

The indices $a, b = 1, \ldots, 2N_{\text{s}}$ run over the $2N_{\text{s}}$ eigenvalues of the BdG Hamiltonian and $N_{\text{s}} = N_{\text{c}}N_{\text{orb}}$ is the number of lattice sites (see Fig. 2 in the main text). The square bracket notation $[M]_{a,b}$ denotes the matrix element in row a and column b of the matrix M while $\delta_{a,b}$ is the Kronecker delta. The eigenvalues $E_a(\mathbf{q})$ of the BdG Hamiltonian depend on $\mathbf{q}$ either directly or indirectly in the same way as the grand potential. The notation $E_a(\mathbf{q})$ is just a convenient short-hand for the right hand side of Eq. (9).

The canonical transformation of the fermionic operators $\hat{\mathbf{c}}$

$$\hat{\mathbf{c}} = \mathcal{W}(\mathbf{q})\hat{\boldsymbol{\gamma}}\,, \tag{10}$$

preserves the anticommutation relations. The operators $\hat{\boldsymbol{\gamma}} = (\gamma_1, \ldots, \gamma_{2N_{\text{s}}})^T$ are therefore annihilation or creation operators of the fermionic quasiparticles that are the elementary excitations above the ground state of the mean-field Hamiltonian. Using the canonical transformation (10) the trace on the right hand side of Eq. (7) can be evaluated

$$\text{Tr}[e^{-\beta \hat{\mathbf{c}}^\dagger H_{\text{BdG}}(\mathbf{q})\hat{\mathbf{c}}}] = \text{Tr}[e^{-\beta\hat{\boldsymbol{\gamma}}^\dagger E(\mathbf{q})\hat{\boldsymbol{\gamma}}}] = \prod_a \text{Tr}[e^{-\beta E_a(\mathbf{q})\hat{\gamma}_a^\dagger\hat{\gamma}_a}] = \prod_a (1 + e^{-\beta E_a(\mathbf{q})})\,. \tag{11}$$

The above identity is a consequence of the fact that the number operators $\hat{\gamma}_a^\dagger\hat{\gamma}_a$ are mutually commuting and that the trace of a tensor product is the product of traces $\text{Tr}[A\otimes B] = \text{Tr}[A]\text{Tr}[B]$. The grand potential thus reads

$$\Omega\big(T, \mu(\mathbf{q}), \Delta_{\mathbf{i}}(\mathbf{q}), \mathbf{q}\big) = \text{Tr}\, H_\downarrow(\mathbf{q}) + \sum_{\mathbf{i}} \frac{|\Delta_{\mathbf{i}}(\mathbf{q})|^2}{U} - \frac{1}{\beta}\sum_a \ln(1 + e^{-\beta E_a(\mathbf{q})})\,. \tag{12}$$

At equilibrium the grand potential attains a minimum, therefore it must be stationary with respect to variations of the pairing potential, namely

$$0 = \frac{\partial\Omega}{\partial\Delta_{\mathbf{i}}^*} = \frac{\Delta_{\mathbf{i}}}{U} + \text{Tr}[\hat{\rho}_{\text{th}}\hat{c}_{\mathbf{i}\downarrow}\hat{c}_{\mathbf{i}\uparrow}] = \frac{\Delta_{\mathbf{i}}}{U} + \langle\hat{c}_{\mathbf{i}\downarrow}\hat{c}_{\mathbf{i}\uparrow}\rangle\,. \tag{13}$$

The expectation values $\langle\,\cdot\,\rangle = \text{Tr}[\hat{\rho}_{\text{th}}\,\cdot\,]$ are taken with respect to the equilibrium state described by the thermal density matrix $\hat{\rho}_{\text{th}} = e^{-\beta\hat{\mathcal{H}}_{\text{m.f.}}}/\mathcal{Z}_\Omega$. Eq. (13) is the equation that guarantees the self-consistency of the mean-field approximation and is written in the most general form valid for an inhomogeneous system. The total particle number N can be fixed using the thermodynamic relation

$$N = -\frac{\partial\Omega}{\partial\mu} = \sum_{\mathbf{i}}\langle\hat{c}_{\mathbf{i}\uparrow}^\dagger\hat{c}_{\mathbf{i}\uparrow} + \hat{c}_{\mathbf{i}\downarrow}^\dagger\hat{c}_{\mathbf{i}\downarrow}\rangle\,. \tag{14}$$

Eqs. (13)-(14) must in general be solved simultaneously by numerical means. Suppose that such a solution has been found for a given $\mathbf{q}$. We now want to show that a self-consistent solution of the time-reversal transformed state with $\mathbf{q}\to-\mathbf{q}$ can be immediately obtained according to the transformation

$$\Delta_{\mathbf{i}}(\mathbf{q}) = \Delta_{\mathbf{i}}^*(-\mathbf{q})\,, \tag{15}$$

$$\mu(\mathbf{q}) = \mu(-\mathbf{q})\,. \tag{16}$$

The proof proceeds by first solving the BdG Hamiltonian $H_{\mathrm{BdG}}(-\mathbf{q})$ assuming the above relations and then showing that indeed the self-consistency equations (13) and (14) are satisfied. Eqs. (15)-(16) imply that the BdG Hamiltonian has the following symmetry property

$$t_y H_{\mathrm{BdG}}(\mathbf{q}) t_y = -H_{\mathrm{BdG}}(-\mathbf{q}) \quad \text{with} \quad t_y = \begin{pmatrix} 0 & -i \\ i & 0 \end{pmatrix} \otimes \mathbf{1}_{N_s}\,. \tag{17}$$

The symmetry operator t_y is simply a Pauli matrix that acts on the particle-hole space of the BdG Hamiltonian, namely the matrix blocks in Eq. (4), and acts as the identity on the orbital degrees of freedom (lattice sites). For $\mathbf{q} = 0$ this symmetry has the properties

$$t_y H t_y^{-1} = -H \quad t_y t_y^{\dagger} = 1 \quad t_y^2 = 1\,. \tag{18}$$

and is called a *chiral* or *sublattice* symmetry according to the terminology of Ref. 1, but it is not related to a particular transformation of the lattice in our case. The symmetry (17) is the result of the particle-hole symmetry which is always present in a BdG Hamiltonian together with time-reversal symmetry (TRS) and invariance with respect to rotations of the spin in the plane perpendicular to the z axis. TRS is broken for $\mathbf{q} \neq 0$ as discussed before. This symmetry implies that for $\mathbf{q} = 0$ our system belongs to the AIII (chiral unitary) symmetry class, while for $\mathbf{q} \neq 0$ the system belongs to the A (unitary) symmetry class[1].

The symmetry in Eq. (17) implies that

$$t_y \mathcal{W}(\mathbf{q}) t_y = \mathcal{W}(-\mathbf{q}) \quad \text{and} \quad t_y E(-\mathbf{q}) t_y = -E(\mathbf{q})\,. \tag{19}$$

It is important to note that the diagonalization of the BdG Hamiltonian $H_{\mathrm{BdG}}(\mathbf{q})$ is not uniquely defined and we can choose to permute the eigenvalues according to the transformation $E(\mathbf{q}) \to PE(\mathbf{q})P$ and $\mathcal{W}(\mathbf{q}) \to \mathcal{W}(\mathbf{q})P$ with P a permutation matrix. However if we require the matrix elements of $\mathcal{W}(\mathbf{q})$ and $E(\mathbf{q})$ to be continuous and differentiable functions of $\mathbf{q}$ then the choice (19) is the only possible one.

In the following we make use of the following additional matrix notations. A diagonal matrix $D = \mathrm{diag}(d_a)$ is completely characterized by the set of its diagonal matrix elements $\{d_a\}_{a=1,\dots,\dim(D)}$. A generic function f of a diagonal matrix $D = \mathrm{diag}(d_a)$ is taken element by element $f(D) = \mathrm{diag}(f(d_a))$ and a function of a Hermitian matrix $H = UDU^{\dagger}$, with U the unitary matrix that diagonalizes H, is defined by $f(H) = Uf(D)U^{\dagger}$. In particular we use the modulus of an Hermitian matrix $|H| = U|D|U^{\dagger}$ and the sign function $\mathrm{sign}(H) = U\,\mathrm{sign}(D)\,U^{\dagger}$. The sign function is defined in a such a way that $\mathrm{sign}(H)|H| = H$.

We can use the above identities (19) to calculate the expectation values of bilinear combinations of the fermionic operators $\hat{c}_{\mathbf{i}\sigma}, \hat{c}_{\mathbf{i}\sigma}^{\dagger}$ which can be conveniently organized into a matrix

$$\begin{aligned}
\langle \hat{\mathbf{c}} \otimes \hat{\mathbf{c}}^{\dagger} \rangle_{-\mathbf{q}} &= \begin{pmatrix} \langle \hat{c}_{\mathbf{i}\uparrow} \hat{c}_{\mathbf{j}\uparrow}^{\dagger} \rangle_{-\mathbf{q}} & \langle \hat{c}_{\mathbf{i}\uparrow} \hat{c}_{\mathbf{j}\downarrow} \rangle_{-\mathbf{q}} \\ \langle \hat{c}_{\mathbf{i}\downarrow}^{\dagger} \hat{c}_{\mathbf{j}\uparrow}^{\dagger} \rangle_{-\mathbf{q}} & \langle \hat{c}_{\mathbf{i}\downarrow}^{\dagger} \hat{c}_{\mathbf{j}\downarrow} \rangle_{-\mathbf{q}} \end{pmatrix} = \mathcal{W}(-\mathbf{q}) \langle \hat{\boldsymbol{\gamma}} \otimes \hat{\boldsymbol{\gamma}}^{\dagger} \rangle_{-\mathbf{q}} \mathcal{W}^{\dagger}(-\mathbf{q}) = \mathcal{W}(-\mathbf{q}) \frac{1}{e^{-\beta E(-\mathbf{q})} + 1} \mathcal{W}^{\dagger}(-\mathbf{q}) \\
&= \mathbf{1} - t_y \mathcal{W}(\mathbf{q}) \frac{1}{e^{-\beta E(\mathbf{q})} + 1} \mathcal{W}^{\dagger}(\mathbf{q}) t_y = \mathbf{1} - t_y \langle \hat{\mathbf{c}} \otimes \hat{\mathbf{c}}^{\dagger} \rangle_{\mathbf{q}} t_y = \begin{pmatrix} \langle \hat{c}_{\mathbf{j}\downarrow} \hat{c}_{\mathbf{i}\downarrow}^{\dagger} \rangle_{\mathbf{q}} & \langle \hat{c}_{\mathbf{i}\downarrow}^{\dagger} \hat{c}_{\mathbf{j}\uparrow}^{\dagger} \rangle_{\mathbf{q}} \\ \langle \hat{c}_{\mathbf{i}\uparrow} \hat{c}_{\mathbf{j}\downarrow} \rangle_{\mathbf{q}} & \langle \hat{c}_{\mathbf{j}\uparrow}^{\dagger} \hat{c}_{\mathbf{i}\uparrow} \rangle_{\mathbf{q}} \end{pmatrix}.
\end{aligned} \tag{20}$$

Since the quasiparticle operators $\hat{\gamma}_a$ diagonalize the BdG Hamiltonian $H_{\mathrm{BdG}}(\mathbf{q})$ the matrix $\langle \hat{\boldsymbol{\gamma}} \otimes \hat{\boldsymbol{\gamma}}^{\dagger} \rangle_{\mathbf{q}}$ is diagonal

$$\langle \hat{\boldsymbol{\gamma}} \otimes \hat{\boldsymbol{\gamma}}^{\dagger} \rangle_{\mathbf{q}} = \mathrm{diag}\left(\frac{1}{e^{-\beta E_a(\mathbf{q})} + 1} \right) = \frac{1}{e^{-\beta E(\mathbf{q})} + 1}\,. \tag{21}$$

Specializing the result in Eq. (20) for $\mathbf{i} = \mathbf{j}$ one obtains Eqs. (15). Moreover since the total particle number $N = \sum_{\mathbf{i}} (n_{\mathbf{i}\uparrow} + n_{\mathbf{i}\downarrow}) = \sum_{\mathbf{i}} \langle \hat{c}_{\mathbf{i}\uparrow}^{\dagger} \hat{c}_{\mathbf{i}\uparrow} + \hat{c}_{\mathbf{i}\downarrow}^{\dagger} \hat{c}_{\mathbf{i}\downarrow} \rangle$ is unchanged also the chemical potential $\mu(\mathbf{q})$ is an even function, validating Eq. (16). This means that from a self-consistent solution for a certain value of $\mathbf{q}$ we

can immediately provide a self-consistent solution for the TRS conjugate ground state $\mathbf{q} \to -\mathbf{q}$. Eq. (20) shows that in principle it is possible to have a nonzero spin density in a state with finite current. From Eq. (20) the density of the z component of the spin $S_z(\mathbf{q}) = n_{\mathbf{i}\uparrow}(\mathbf{q}) - n_{\mathbf{i}\downarrow}(\mathbf{q}) = \langle \hat{c}^\dagger_{\mathbf{i}\uparrow}\hat{c}_{\mathbf{i}\uparrow}\rangle_{\mathbf{q}} - \langle \hat{c}^\dagger_{\mathbf{i}\downarrow}\hat{c}_{\mathbf{i}\downarrow}\rangle_{\mathbf{q}}$ is an odd function of $\mathbf{q}$, which is consistent with TRS.

The properties (15)-(16), imply that the grand potential is an even function of $\mathbf{q}$. In fact, consider only the first and the last term in Eq. (12), since the remaining term is an even function of $\mathbf{q}$ according to our previous discussion,

$$\begin{aligned}\mathrm{Tr}\, H^{\downarrow}(-\mathbf{q}) - \frac{1}{\beta}\sum_a \ln(1+e^{-\beta E_a(-\mathbf{q})}) = \mathrm{Tr}\, H^{\uparrow}(\mathbf{q}) - \frac{1}{\beta}\sum_a \ln(1+e^{-\beta(-E_a(\mathbf{q}))})\\ = \mathrm{Tr}\, H^{\uparrow}(\mathbf{q}) - \mathrm{Tr} H_{\mathrm{BdG}}(\mathbf{q}) - \frac{1}{\beta}\sum_a \ln(1+e^{-\beta E_a(\mathbf{q})}) = \mathrm{Tr}\, H^{\downarrow}(\mathbf{q}) - \frac{1}{\beta}\sum_a \ln(1+e^{-\beta E_a(\mathbf{q})})\,.\end{aligned} \tag{22}$$

This implies that $\mathbf{q} = 0$ is a stationary point of the grand potential, a candidate ground state. In order to check the stability of this stationary point it is necessary to calculate the second derivative with respect to $\mathbf{q}$. This amounts to the evaluation of the *superfluid weight* D_{s}. The superfluid weight is defined as the second derivative of the *free energy* $F(T, N, \Delta_{\mathbf{i}}(\mathbf{q}), \mathbf{q}) = \Omega(T, \mu(\mathbf{q}), \Delta_{\mathbf{i}}(\mathbf{q}), \mathbf{q}) + N\mu(\mathbf{q})$ since the total particle number is constant,

$$[D_{\mathrm{s}}]_{i,j} = \frac{1}{V\hbar^2}\frac{\partial^2 F}{\partial q_i \partial q_j} = \frac{1}{V\hbar^2}\frac{\partial^2(\Omega + \mu N)}{\partial q_i \partial q_j}\,, \tag{23}$$

where V is the system volume (in two dimensions the area A is used instead). In general the superfluid weight in an anisotropic system depends on the direction and is thus a tensor. As emphasised in our notation the quasiparticle energies and thus the grand potential depend on $\mathbf{q}$ both through $\mu(\mathbf{q})$ and $\Delta(\mathbf{q})$ and directly through the diagonal terms of the BdG Hamiltonian. It will be now shown that in fact it is not necessary to know the dependence of $\mu(\mathbf{q}), \Delta(\mathbf{q})$ to calculate the superfluid density and only their $\mathbf{q} = 0$ value is needed. This result has been proved in Ref. 2, but we repeat the proof here with some modifications. By a suitable gauge transformation $\hat{c}_{\mathbf{i}\sigma} \to e^{i\phi_{\mathbf{i}}(\mathbf{q})}\hat{c}_{\mathbf{i}\sigma}$ it is possible to take $\Delta_{\mathbf{i}}(\mathbf{q})$ real and at the same time preserving the property (15). This gauge will be used in the following. Taking the total derivative of the free energy with respect to $\mathbf{q}$ gives

$$\frac{\partial F}{\partial q_i} = \sum_{\mathbf{i}} \left.\frac{\partial \Omega}{\partial \Delta_{\mathbf{i}}}\right|_{\mu,\mathbf{q}} \frac{\partial \Delta_{\mathbf{i}}}{\partial q_i} + \left(\left.\frac{\partial \Omega}{\partial \mu}\right|_{\Delta_{\mathbf{i}},\mathbf{q}} + N\right)\frac{\partial \mu}{\partial q_i} + \left.\frac{\partial \Omega}{\partial q_i}\right|_{\Delta_{\mathbf{i}},\mu} = \left.\frac{\partial \Omega}{\partial q_i}\right|_{\Delta_{\mathbf{i}},\mu}\,. \tag{24}$$

This result is valid for arbitrary $\mathbf{q}$ and it relies on the fact that μ and $\Delta_{\mathbf{i}}$ satisfy Eqs. (13)-(14), i.e. they are self-consistent solutions. As a consequence a stationary point of the free energy corresponds to the condition $\partial\Omega/\partial q_i|_{\Delta_{\mathbf{i}},\mu} = 0$ on the grand potential which is satisfied for $\mathbf{q} = 0$ as we have seen above. From Eqs. (15)-(16) and the fact that $\Delta_{\mathbf{i}}(\mathbf{q})$ is real, one has $\partial\Delta_{\mathbf{i}}/\partial q_i = \partial\mu/\partial q_i = 0$ at $\mathbf{q} = 0$. This last result is useful when taking one more derivative of the free energy and evaluating it at equilibrium

$$\left.\frac{\partial^2 F}{\partial q_j \partial q_i}\right|_{\mathbf{q}=0} = \sum_{\mathbf{i}} \left.\frac{\partial^2 \Omega}{\partial \Delta_{\mathbf{i}}\partial q_i}\frac{\partial \Delta_{\mathbf{i}}}{\partial q_j}\right|_{\mathbf{q}=0} + \left.\frac{\partial^2 \Omega}{\partial \mu \partial q_i}\frac{\partial \mu}{\partial q_j}\right|_{\mathbf{q}=0} + \left.\frac{\partial \Omega}{\partial q_j \partial q_i}\right|_{\mathbf{q}=0} = \left.\frac{\partial \Omega}{\partial q_j \partial q_i}\right|_{\mathbf{q}=0}\,. \tag{25}$$

In the above equation the partial derivatives of the grand potential with respect to $\Delta_{\mathbf{i}}, \mu, q_i$ are always taken keeping the other quantities fixed (introducing a notation to specify this would be quite cumbersome). The derivatives of the free energy with respect to q_i instead are total derivatives in the sense that the dependence of $\mu(\mathbf{q}), \Delta(\mathbf{q})$ on $\mathbf{q}$ needs to be taken into account and the corresponding derivatives performed. The final result is then

$$[D_{\mathrm{s}}]_{i,j} = \frac{1}{V\hbar^2}\left.\frac{\partial^2 \Omega}{\partial q_i \partial q_j}\right|_{\Delta_{\mathbf{i}},\mu,\mathbf{q}=0}\,. \tag{26}$$

The important point is that the above derivatives are taken for $\Delta_{\mathbf{i}}, \mu$ constant even if they are themselves functions of $\mathbf{q}$. Only the value of the order parameters at $\mathbf{q} = 0$ is needed to calculate the superfluid weight. This is unsurprising since the superfluid weight is a linear response coefficient and therefore it depends only on the properties of the ground state.

Supplementary Note 2: Wannier functions and tight-binding Hamiltonian

Consider the Schrödinger equation with a periodic potential $V(\mathbf{r}) = V(\mathbf{r} + \mathbf{a}_i)$, with $\mathbf{a}_i$ the fundamental vectors that define the Bravais lattice,

$$H\psi(\mathbf{r}) = \left(-\frac{\hbar^2}{2m}\boldsymbol{\nabla}^2 + V(\mathbf{r})\right)\psi(\mathbf{r}) = \varepsilon\psi(\mathbf{r})\,. \tag{27}$$

Bloch theorem states that the solutions can be labelled by a band index n and a quasimomentum index $\mathbf{k}$ and take the form[3]

$$\psi_{n\mathbf{k}}(\mathbf{r}) = e^{i\mathbf{k}\cdot\mathbf{r}}g_{n\mathbf{k}}(\mathbf{r})\,, \quad \text{with eigenvalue} \quad \varepsilon_{n\mathbf{k}}\,. \tag{28}$$

The functions $g_{n\mathbf{k}}(\mathbf{r})$, called periodic Bloch functions (Bloch functions for brevity), are periodic in the real space coordinate $g_{n\mathbf{k}}(\mathbf{r}) = g_{n\mathbf{k}}(\mathbf{r} + \mathbf{a}_i)$. The eigenvalues are periodic in the quasimomentum $\varepsilon_{n\mathbf{k}} = \varepsilon_{n(\mathbf{k}+\mathbf{d}_i)}$, with $\mathbf{d}_i$ the fundamental vectors of the reciprocal lattice that are defined by $\mathbf{a}_i \cdot \mathbf{d}_j = 2\pi\delta_{ij}$. We adopt the convention that the periodic Bloch functions are normalized $\int_\Omega d^3\mathbf{r}\,|g_{n\mathbf{k}}(\mathbf{r})|^2 = 1$, where the symbol $\int_\Omega d^3\mathbf{r}$ denotes the integration over one unit cell. The band index n runs from 1 to $+\infty$ since there are an infinite number of bands in the continuum. Consider now a subset $\mathcal{S}$ of relevant bands, also called *composite band*, separated from other bands below and above by sufficiently large bands gaps. It is possible to construct a particular convenient basis of localized functions that span exactly the same subspace spanned by the Bloch plane waves $\psi_{n\mathbf{k}}$ relative to the composite band. These are called Wannier functions and are defined by

$$\begin{aligned} w_\alpha(\mathbf{r} - \mathbf{r_i}) &= \frac{V_\Omega}{(2\pi)^3}\int d^3\mathbf{k}\, e^{-i\mathbf{k}\cdot\mathbf{r_i}} \sum_{n\in\mathcal{S}} [U_{\mathbf{k}}]_{\alpha,n}\psi_{n\mathbf{k}}(\mathbf{r}) \\ &= \frac{V_\Omega}{(2\pi)^3}\int d^3\,\mathbf{k} e^{i\mathbf{k}\cdot(\mathbf{r}-\mathbf{r_i})} \sum_{n\in\mathcal{S}} [U_{\mathbf{k}}]_{\alpha,n} g_{n\mathbf{k}}(\mathbf{r}) \\ &= \frac{V_\Omega}{(2\pi)^3}\int d^3\,\mathbf{k} e^{i\mathbf{k}\cdot(\mathbf{r}-\mathbf{r_i})} \sum_{n\in\mathcal{S}} [U_{\mathbf{k}}]_{\alpha,n} g_{n\mathbf{k}}(\mathbf{r} - \mathbf{r_i})\,. \end{aligned} \tag{29}$$

V_Ω is the volume of the unit cell (in two dimension the area A_Ω of the unit cell is used instead) and the vector $\mathbf{r_i} = i_x\mathbf{a}_1 + i_y\mathbf{a}_2 + i_z\mathbf{a}_3$, labelled by a triplet of integers $\mathbf{i} = (i_x, i_y, i_z)^T$, is a generic vector of the lattice. In the last line of the above equation we have made use of the fact that $g_{n\mathbf{k}}(\mathbf{r}) = g_{n\mathbf{k}}(\mathbf{r} - \mathbf{r_i})$ for arbitrary $\mathbf{i}$. The matrix $U_{\mathbf{k}}$ is a $\mathbf{k}$-dependent unitary matrix that has to be chosen in order to obtain properly localized Wannier functions.

The Wannier functions $w_\alpha(\mathbf{r} - r_i)$ in Eq. (29) are obtained by the Fourier transform of the Bloch functions $\psi_{n\mathbf{k}}(\mathbf{r})$. The Fourier transform is a unitary operator, therefore the orthonormality of the Bloch functions is inherited by the Wannier functions

$$\int d^3\mathbf{r}\,\psi^*_{n\mathbf{k}}(\mathbf{r})\psi_{n'\mathbf{k}'}(\mathbf{r}) = \frac{(2\pi)^3}{V_\Omega}\delta_{n,n'}\delta(\mathbf{k} - \mathbf{k}') \Rightarrow \int d^3\mathbf{r}\, w^*_\alpha(\mathbf{r} - \mathbf{r_i})w_\beta(\mathbf{r} - \mathbf{r_j}) = \delta_{\alpha,\beta}\delta_{\mathbf{i},\mathbf{j}}\,. \tag{30}$$

According to Ref. 4 if the Chern numbers (one number in 2D and three numbers in 3D) of the composite band are zero then it is possible to choose $U_{\mathbf{k}}$ is such a way that the Wannier functions are exponentially localized. Moreover there are practical methods to calculate the matrices $U_{\mathbf{k}}$ that guarantee that the Wannier functions are maximally localized, more precisely they minimize a suitable localization functional (see Supplementary Note 5). The Wannier functions are useful since they are a basis of states for the

subspace of the Hilbert space relative to the given composite band and are very simple since they are translated copies by displacements $\mathbf{r_i}$ of the small set of functions $\{w_\alpha(\mathbf{r})\}$. The centers $\mathbf{r}_{\mathbf{i}\alpha}$ of the Wannier functions are defined as

$$\mathbf{r}_{\mathbf{i}\alpha} = i_x\mathbf{a}_1 + i_y\mathbf{a}_2 + i_z\mathbf{a}_3 + \mathbf{b}_\alpha\,, \quad \text{with} \quad \mathbf{b}_\alpha = \int d^3\mathbf{r}\,|w_\alpha(\mathbf{r})|^2\mathbf{r}\,. \tag{31}$$

We now consider the Hamiltonian in second quantized form in the basis of Wannier functions. The braket notation is used for the Wannier functions $\langle\mathbf{r}|\mathbf{i}\alpha\rangle = w_\alpha(\mathbf{r}-\mathbf{r_i})$. The second quantized quadratic Hamiltonian is $\hat{\mathcal{H}} = \int d^3\mathbf{r}\,\hat{\psi}^\dagger(\mathbf{r})H\hat{\psi}(\mathbf{r})$ with $\hat{\psi}(\mathbf{r})$ the fermionic field operator satisfying the usual anticommutation relations $\{\hat{\psi}(\mathbf{r}),\hat{\psi}^\dagger(\mathbf{r}')\} = \delta(\mathbf{r}-\mathbf{r}')$. We expand the field operator in the basis of Wannier functions

$$\hat{\psi}(\mathbf{r}) = \sum_{\mathbf{i}}\sum_{\alpha\in\mathcal{S}} w_\alpha(\mathbf{r}-\mathbf{r_i})\hat{c}_{\mathbf{i}\alpha} + \sum_{\mathbf{i}}\sum_{\gamma\in\bar{\mathcal{S}}} \bar{w}_\gamma(\mathbf{r}-\mathbf{r_i})\hat{d}_{\mathbf{i}\gamma}\,. \tag{32}$$

The functions $\bar{w}_\gamma(\mathbf{r}-\mathbf{r_i})$ are Wannier functions of the bands not included in the chosen composite band, and belong to the complementary set $\bar{\mathcal{S}}$. Inserting this expansion into the expression for $\hat{\mathcal{H}}$ one obtains

$$\begin{aligned}\hat{\mathcal{H}} = \int d^3\mathbf{r}\,\hat{\psi}^\dagger(\mathbf{r})H\hat{\psi}(\mathbf{r}) &= \sum_{\alpha,\beta\in\mathcal{S}}\sum_{\mathbf{i},\mathbf{j}} \hat{c}^\dagger_{\mathbf{i}\alpha}\langle\mathbf{i}\alpha|\,H\,|\mathbf{j}\beta\rangle\,\hat{c}_{\mathbf{j}\beta} + \sum_{\gamma,\delta\in\bar{\mathcal{S}}}\sum_{\mathbf{i},\mathbf{j}} \hat{d}^\dagger_{\mathbf{i}\gamma}\langle\mathbf{i}\gamma|\,H\,|\mathbf{j}\delta\rangle\,\hat{d}_{\mathbf{j}\delta} \\ &= \sum_{\alpha,\beta\in\mathcal{S}}\sum_{\mathbf{i},\mathbf{j}} \hat{c}^\dagger_{\mathbf{i}\alpha}K_{\mathbf{i}\alpha,\mathbf{j}\beta}\hat{c}_{\mathbf{j}\beta} + \sum_{\gamma,\delta\in\bar{\mathcal{S}}}\sum_{\mathbf{i},\mathbf{j}} \hat{d}^\dagger_{\mathbf{i}\gamma}\bar{K}_{\mathbf{i}\gamma,\mathbf{j}\delta}\hat{d}_{\mathbf{j}\delta}\,.\end{aligned} \tag{33}$$

The key point in the above equation is that matrix elements of the form $\langle\mathbf{i}\alpha|\,H\,|\mathbf{j}\gamma\rangle$ between a Wannier function belonging to the composite band ($\alpha\in\mathcal{S}$) and a Wannier function in the complementary set ($\gamma\in\bar{\mathcal{S}}$) vanish, the reason being that they are built from eigenfunctions of the Hamiltonian that belong to orthogonal subspaces. On the other hand off-diagonal matrix elements $\langle\mathbf{i}\alpha|\,H\,|\mathbf{j}\beta\rangle$ with $\alpha,\beta\in\mathcal{S}$ are in general nonzero due to the fact that the Wannier functions are not eigenstates of the Hamiltonian. In particular there is a coupling between Wannier functions with $\alpha\neq\beta$ due to the mixing given by the unitary matrix $U_\mathbf{k}$ in the definition (29). From the definition (33) it is also evident that the hopping matrix elements are translationally invariant $K_{\mathbf{i}\alpha,\mathbf{j}\beta} = K_{\alpha,\beta}(\mathbf{i}-\mathbf{j})$. For exponentially localized Wannier function the matrix elements $K_{\alpha,\beta}(\mathbf{i}-\mathbf{j})$ decay rapidly with the distance $\mathbf{i}-\mathbf{j}$ between unit cells and it is usually a good approximation to retain only the matrix elements between a limited number of neighbouring Wannier functions. The result is the so-called tight-binding Hamiltonian[3] which provides the physical picture of particles moving by discrete tunneling events between the sites of the lattice.

Finally Eqs. (28)-(29) in the main text can be obtained by expanding the field operators in plane waves $\hat{c}_{\mathbf{i}\alpha\sigma} = \frac{1}{\sqrt{N_\mathrm{c}}}\sum_\mathbf{k} e^{i\mathbf{k}\cdot\mathbf{r}_{\mathbf{i}\alpha}}\hat{c}_{\alpha\mathbf{k}\sigma}$ (N_c is the number of unit cells)

$$\begin{aligned}\sum_{\alpha,\beta\in\mathcal{S}}\sum_{\mathbf{i},\mathbf{j}} \hat{c}^\dagger_{\mathbf{i}\alpha\sigma}K^\sigma_{\mathbf{i}\alpha,\mathbf{j}\beta}\hat{c}_{\mathbf{j}\beta\sigma} &= \frac{1}{N_\mathrm{c}}\sum_{\alpha,\beta\in\mathcal{S}}\sum_{\mathbf{i},\mathbf{j}}\left(\sum_{\mathbf{k}_1} e^{-i\mathbf{k}_1\cdot\mathbf{r}_{\mathbf{i}\alpha}}\hat{c}^\dagger_{\alpha\mathbf{k}_1\sigma}\right)K^\sigma_{\mathbf{i}\alpha,\mathbf{j}\beta}\left(\sum_{\mathbf{k}_2} e^{i\mathbf{k}_2\cdot\mathbf{r}_{\mathbf{j}\beta}}\hat{c}_{\beta\mathbf{k}_2\sigma}\right) \\ &= \sum_{\alpha,\beta\in\mathcal{S}}\sum_{\mathbf{k}_1,\mathbf{k}_2} e^{-i\mathbf{k}_1\cdot\mathbf{b}_\alpha}\hat{c}^\dagger_{\alpha\mathbf{k}_1\sigma}e^{i\mathbf{k}_2\cdot\mathbf{b}_\beta}\hat{c}_{\beta\mathbf{k}_2\sigma}\left(\frac{1}{N_\mathrm{c}}\sum_{\mathbf{j}} e^{i(\mathbf{k}_2-\mathbf{k}_1)\cdot\mathbf{r_j}}\right)\sum_{\mathbf{i}-\mathbf{j}} e^{-i\mathbf{k}_1\cdot\mathbf{r}_{\mathbf{i}-\mathbf{j}}}K^\sigma_{\alpha,\beta}(\mathbf{i}-\mathbf{j}) \\ &= \sum_{\alpha,\beta\in\mathcal{S}}\sum_{\mathbf{k}} \hat{c}^\dagger_{\alpha\mathbf{k}\sigma}\hat{c}_{\beta\mathbf{k}\sigma}\left(\sum_{\mathbf{i}-\mathbf{j}} e^{-i\mathbf{k}\cdot(\mathbf{r}_{\mathbf{i}\alpha}-\mathbf{r}_{\mathbf{j}\beta})}K^\sigma_{\mathbf{i}\alpha,\mathbf{j}\beta}\right) = \sum_{\alpha,\beta\in\mathcal{S}}\sum_{\mathbf{k}} \hat{c}^\dagger_{\alpha\mathbf{k}\sigma}[\widetilde{K}^\sigma(\mathbf{k})]_{\alpha,\beta}\hat{c}_{\beta\mathbf{k}\sigma}\,.\end{aligned} \tag{34}$$

Supplementary Note 3: Superfluid weight in a multiband system at finite temperature

From now on we consider a translational invariant Hamiltonian and we use the notation employed in the main text. The symmetry constrain in Eq. (19) implies that for $\mathbf{q}=0$ the matrices $E_\mathbf{k}(\mathbf{q})$ and $\mathcal{W}_\mathbf{k}(\mathbf{q})$ have

the form shown in Eqs. (8) and (9) in the main text. It is convenient to rewrite the grand potential in Eq. (12) in a slightly different form which can be derived in a way similar to Eq. (22)

$$\Omega(T,\mu,\Delta,\mathbf{q}) = \sum_{\mathbf{k}} \frac{1}{2}\big(\mathrm{Tr}\, H^{\uparrow}_{\mathbf{k}}(\mathbf{q}) + \mathrm{Tr}\, H^{\uparrow}_{\mathbf{k}}(-\mathbf{q})\big) - \frac{1}{2\beta}\sum_{\mathbf{k}} \mathrm{Tr}\left[\ln 2\big(1+\cosh\beta H_{\mathbf{k}}(\mathbf{q})\big)\right] + N_c \frac{\mathrm{Tr}|\Delta(\mathbf{q})|^2}{U}\,. \quad (35)$$

According to the results in the previous section the last term $\mathrm{Tr}|\Delta(\mathbf{q})|^2/U = \sum_\alpha |\Delta_\alpha(\mathbf{q})|^2/U$ can be dropped since it does not contribute to the superfluid density. Also the first term $\frac{1}{2}\big(\mathrm{Tr}\, H^{\uparrow}(\mathbf{q}) + \mathrm{Tr}\, H^{\uparrow}(-\mathbf{q})\big) = \frac{1}{2}\sum_{\mathbf{k}}\big(\mathrm{Tr}\left[\varepsilon_{\mathbf{k}-\mathbf{q}} - \mu\mathbf{1}\right] + \mathrm{Tr}\left[\varepsilon_{\mathbf{k}+\mathbf{q}} - \mu\mathbf{1}\right]\big)$ does not contribute in the thermodynamic limit since $\varepsilon_{\mathbf{k}}$ is a periodic function on the Brillouin zone. However we keep this term for reasons that will be clear in the following. Note that in the low temperature limit $\beta \to +\infty$ the second term reduces to $-\frac{1}{2}\mathrm{Tr}\left[|H_{\mathbf{k}}(\mathbf{q})|\right]$ in agreement with Eq. (10) in the main text.

The first derivative of the grand potential with respect to $\mathbf{q}$ is the current density

$$\mathbf{J}(\mathbf{q}) = \frac{1}{V\hbar}\frac{\partial\Omega}{\partial\mathbf{q}}\bigg|_{\mu,\Delta} = \frac{1}{V\hbar}\sum_{\mathbf{k}}\left(\frac{1}{2}\left(\mathrm{Tr}[\partial_{\mathbf{k}}\varepsilon_{\mathbf{k}+\mathbf{q}}] - \mathrm{Tr}[\partial_{\mathbf{k}}\varepsilon_{\mathbf{k}-\mathbf{q}}]\right) - \frac{1}{2}\mathrm{Tr}\left[\tanh\left(\frac{\beta E_{\mathbf{k}}(\mathbf{q})}{2}\right)\partial_{\mathbf{q}}E_{\mathbf{k}}(\mathbf{q})\right]\right). \quad (36)$$

The matrix elements of the diagonal matrix $\partial_{\mathbf{q}}E_{\mathbf{k}}(\mathbf{q})$ can be calculated using the Hellman-Feynman theorem and the expression for $H_{\mathbf{k}}(\mathbf{q})$ in Eq. (6) in the main text

$$[\partial_{\mathbf{q}}E_{\mathbf{k}}(\mathbf{q})]_{a,a} = \Big[\mathcal{W}^\dagger_{\mathbf{k}}(\mathbf{q})\,\partial_{\mathbf{q}}H_{\mathbf{k}}(\mathbf{q})\,\mathcal{W}_{\mathbf{k}}(\mathbf{q})\Big]_{a,a}, \qquad \text{with} \qquad \partial_{\mathbf{q}}H_{\mathbf{k}}(\mathbf{q}) = -\begin{pmatrix} \partial_{\mathbf{k}}\varepsilon_{\mathbf{k}-\mathbf{q}} & \partial_{\mathbf{q}}\mathcal{D}_{\mathbf{k}}(\mathbf{q}) \\ -\partial_{\mathbf{q}}\mathcal{D}_{\mathbf{k}}(-\mathbf{q}) & \partial_{\mathbf{k}}\varepsilon_{\mathbf{k}+\mathbf{q}}\end{pmatrix}. \quad (37)$$

We have defined $\mathcal{D}_{\mathbf{k}}(\mathbf{q}) = -\mathcal{G}^\dagger_{\mathbf{k}-\mathbf{q}}\Delta\mathcal{G}_{\mathbf{k}+\mathbf{q}} = \mathcal{D}^\dagger_{\mathbf{k}}(-\mathbf{q})$ in the above equation. Notice that the Hellman-Feynman theorem holds only for the diagonal components $a=b$ of the matrices on the left and right hand side as emphasised by the square bracket notation $[\,\cdot\,]_{a,b=a}$. The matrix $\tanh(E_{\mathbf{k}}(\mathbf{q}))$ is itself diagonal and only the diagonal matrix elements in Eq. (37) are needed in the evaluation of the trace in Eq. (36). It is easy to check that the current is an odd function using the identities in Eq. (19). Again one can distinguish a conventional current contribution and a contribution that comes from the off-diagonal terms in $\partial_{\mathbf{q}}H_{\mathbf{k}}(\mathbf{q})$ as in the zero temperature case discussed in the main text. Indeed the two contributions are separately gauge invariant as explained below.

When taking an additional derivative with respect to $\mathbf{q}$ there will be terms proportional to the second derivative of the BdG Hamiltonian $\partial_{q_i}\partial_{q_j}H_{\mathbf{k}}(\mathbf{q})$ and first derivatives of the unitary matrix $\partial_{\mathbf{q}}\mathcal{W}_{\mathbf{k}}(\mathbf{q})$. The following identity is useful in order to express the off-diagonal matrix elements of $\mathcal{W}^\dagger_{\mathbf{k}}(\mathbf{q})\partial_{\mathbf{q}}\mathcal{W}_{\mathbf{k}}(\mathbf{q}) = -\big(\partial_{\mathbf{q}}\mathcal{W}^\dagger_{\mathbf{k}}(\mathbf{q})\big)\mathcal{W}_{\mathbf{k}}(\mathbf{q})$ only in terms of the ground state solution

$$\Big[\mathcal{W}^\dagger_{\mathbf{k}}(\mathbf{q})\partial_{\mathbf{q}}\mathcal{W}_{\mathbf{k}}(\mathbf{q})\Big]_{a,b} = -\frac{\Big[\mathcal{W}^\dagger_{\mathbf{k}}(\mathbf{q})\,\partial_{\mathbf{q}}H_{\mathbf{k}}(\mathbf{q})\,\mathcal{W}_{\mathbf{k}}(\mathbf{q})\Big]_{a,b}}{[E_{\mathbf{k}}(\mathbf{q})]_{a,a} - [E_{\mathbf{k}}(\mathbf{q})]_{b,b}} \quad \text{for} \quad a \neq b\,. \quad (38)$$

The second derivatives of the quasiparticle excitaton energies can therefore be written as

$$\begin{aligned}[\partial_{q_i}\partial_{q_j}E_{\mathbf{k}}(\mathbf{q})]_{a,a} &= \Big[\mathcal{W}^\dagger_{\mathbf{k}}(\mathbf{q})\,\partial_{q_i}\partial_{q_j}H_{\mathbf{k}}(\mathbf{q})\,\mathcal{W}_{\mathbf{k}}(\mathbf{q})\Big]_{a,a} \\ &+ \sum_{\substack{b\\ a\neq b}} \frac{1}{[E_{\mathbf{k}}(\mathbf{q})]_{a,a} - [E_{\mathbf{k}}(\mathbf{q})]_{b,b}}\left(\Big[\mathcal{W}^\dagger_{\mathbf{k}}(\mathbf{q})\,\partial_{q_i}H_{\mathbf{k}}(\mathbf{q})\,\mathcal{W}_{\mathbf{k}}(\mathbf{q})\Big]_{a,b}\Big[\mathcal{W}^\dagger_{\mathbf{k}}(\mathbf{q})\,\partial_{q_j}H_{\mathbf{k}}(\mathbf{q})\,\mathcal{W}_{\mathbf{k}}(\mathbf{q})\Big]_{b,a} + (i\leftrightarrow j)\right).\end{aligned} \quad (39)$$

Taking one more derivative of the current density and setting $\mathbf{q}=0$ leads to the superfluid weight

$$[D_{\mathrm{s}}]_{i,j} = \frac{1}{V\hbar^2}\sum_{\mathbf{k}}\left\{\mathrm{Tr}[\partial_{k_i}\partial_{k_j}\varepsilon_{\mathbf{k}}] - \frac{1}{2}\mathrm{Tr}\left[\tanh\left(\frac{\beta E_{\mathbf{k}}}{2}\right)\partial_{q_i}\partial_{q_j}E_{\mathbf{k}}\right] - \frac{1}{2}\mathrm{Tr}\left[\frac{\beta}{2\cosh^2(\beta E_{\mathbf{k}}/2)}\partial_{q_i}E_{\mathbf{k}}\partial_{q_j}E_{\mathbf{k}}\right]\right\}, \quad (40)$$

where $E_{\mathbf{k}} = E_{\mathbf{k}}(\mathbf{q}=0)$, $\partial_{q_i} E_{\mathbf{k}} = \partial_{q_i} E_{\mathbf{k}}(\mathbf{q}=0)$ and $\partial_{q_i}\partial_{q_j} E_{\mathbf{k}} = \partial_{q_i}\partial_{q_j} E_{\mathbf{k}}(\mathbf{q}=0)$. Eq. (40) can be written in a more convenient form by introducing some definitions (see Eqs. (8)-(9) in the main text)

$$N_{\mathbf{k},i} = \mathcal{W}^\dagger_{\mathbf{k}}(\mathbf{q}=0)\,\partial_{q_i} H_{\mathbf{k}}(\mathbf{q}=0)\,\mathcal{W}_{\mathbf{k}}(\mathbf{q}=0) = \begin{pmatrix} A_{\mathbf{k},i} & B_{\mathbf{k},i} \\ -B_{\mathbf{k},i} & A_{\mathbf{k},i}\end{pmatrix}$$
$$\text{with } \begin{cases} A_{\mathbf{k},i} = \mathcal{U}^\dagger_{\mathbf{k}}\partial_{k_i}\varepsilon_{\mathbf{k}}\mathcal{U}_{\mathbf{k}} + \mathcal{V}^\dagger_{\mathbf{k}}\partial_{k_i}\varepsilon_{\mathbf{k}}\mathcal{V}_{\mathbf{k}} + \mathcal{U}^\dagger_{\mathbf{k}}\partial_{q_i}\mathcal{D}_{\mathbf{k}}\mathcal{V}_{\mathbf{k}} - \mathcal{V}^\dagger_{\mathbf{k}}\partial_{q_i}\mathcal{D}_{\mathbf{k}}\mathcal{U}_{\mathbf{k}} \\ B_{\mathbf{k},i} = \mathcal{U}^\dagger_{\mathbf{k}}\partial_{q_i}\mathcal{D}_{\mathbf{k}}\mathcal{U}_{\mathbf{k}} + \mathcal{V}^\dagger_{\mathbf{k}}\partial_{q_i}\mathcal{D}_{\mathbf{k}}\mathcal{V}_{\mathbf{k}} + \mathcal{V}^\dagger_{\mathbf{k}}\partial_{k_i}\varepsilon_{\mathbf{k}}\mathcal{U}_{\mathbf{k}} - \mathcal{U}^\dagger_{\mathbf{k}}\partial_{k_i}\varepsilon_{\mathbf{k}}\mathcal{V}_{\mathbf{k}} \end{cases} \tag{41}$$

$$[T_{\mathbf{k}}]_{a,b} = \begin{cases} \left[\dfrac{\beta}{2\cosh^2(\beta E_{\mathbf{k}}/2)}\right]_{a,a} & \text{for} \quad a=b\,, \\[2ex] \dfrac{[\tanh(\beta E_{\mathbf{k}}/2)]_{a,a} - [\tanh(\beta E_{\mathbf{k}}/2)]_{b,b}}{[E_{\mathbf{k}}]_{a,a} - [E_{\mathbf{k}}]_{b,b}} & \text{for} \quad a\neq b\,. \end{cases} \tag{42}$$

The abbreviation $\partial_{q_i}\mathcal{D}_{\mathbf{k}} = \partial_{q_i}\mathcal{D}_{\mathbf{k}}(\mathbf{q}=0)$ has just been employed and $\partial_{q_i}\partial_{q_j}\mathcal{D}_{\mathbf{k}} = \partial_{q_i}\partial_{q_j}\mathcal{D}_{\mathbf{k}}(\mathbf{q}=0)$ will be employed in the following. The final result for the superfluid weight tensor contains three contributions

$$[D_{\rm s}]_{i,j} = \frac{1}{V\hbar^2}\frac{\partial^2\Omega}{\partial q_i\partial q_j}\bigg|_{\mu,\Delta,\mathbf{q}=0} = [D_{{\rm s},1}]_{i,j} + [D_{{\rm s},2}]_{i,j} + [D_{{\rm s},3}]_{i,j}\,, \qquad \text{with} \tag{43}$$

$$[D_{{\rm s},1}]_{i,j} = \frac{2}{V\hbar^2}\sum_{\mathbf{k}}\mathrm{Tr}\left[\left(\mathcal{V}_{\mathbf{k}}\frac{1}{e^{-\beta E^>_{\mathbf{k}}}+1}\mathcal{V}^\dagger_{\mathbf{k}} + \mathcal{U}_{\mathbf{k}}\frac{1}{e^{\beta E^>_{\mathbf{k}}}+1}\mathcal{U}^\dagger_{\mathbf{k}}\right)\partial_{k_i}\partial_{k_j}\varepsilon_{\mathbf{k}}\right]\,, \tag{44}$$

$$[D_{{\rm s},2}]_{i,j} = \frac{2}{V\hbar^2}\sum_{\mathbf{k}}\mathrm{Tr}\left[\left(\mathcal{U}_{\mathbf{k}}\mathcal{V}^\dagger_{\mathbf{k}} - \mathcal{U}_{\mathbf{k}}\frac{1}{e^{\beta E^>_{\mathbf{k}}}+1}\mathcal{V}^\dagger_{\mathbf{k}} - \mathcal{V}_{\mathbf{k}}\frac{1}{e^{\beta E^>_{\mathbf{k}}}+1}\mathcal{U}^\dagger_{\mathbf{k}}\right)\partial_{q_i}\partial_{q_j}\mathcal{D}_{\mathbf{k}}\right]\,, \tag{45}$$

$$[D_{{\rm s},3}]_{i,j} = -\frac{1}{2V\hbar^2}\sum_{\mathbf{k}}\sum_{a,b}[T_{\mathbf{k}}]_{a,b}[N_{\mathbf{k},i}]_{a,b}[N_{\mathbf{k},j}]_{b,a}\,. \tag{46}$$

We have introduced the diagonal matrix $E^>_{\mathbf{k}} = \mathrm{diag}(E_{n\mathbf{k}})$, i.e. the upper diagonal block of $E_{\mathbf{k}}$ which contains the positive eigenvalues $E_{n\mathbf{k}} > 0$ (see Eq. (8) in the main text). The $\mathbf{k}$-dependent effective mass tensor $\left[\frac{1}{m_{\mathrm{eff},\mathbf{k}}}\right]_{i,j} = \frac{1}{\hbar^2}\frac{\partial^2\varepsilon_{\mathbf{k}}}{\partial_{k_i}\partial_{k_j}}$ enters the expression of $D_{{\rm s},1}$ which is the combination of the first term in Eq. (40) and the terms proportional to $\frac{\partial^2\varepsilon_{\mathbf{k}}}{\partial_{k_i}\partial_{k_j}}$ in the second term in the same equation (thus our derivation holds also in the case of a finite system for which $\sum_{\mathbf{k}}\mathrm{Tr}[\varepsilon_{\mathbf{k}+\mathbf{q}}]$ is not a constant as a function of $\mathbf{q}$). The analogous quantity that enters $D_{{\rm s},2}$ is

$$\partial_{q_i}\partial_{q_j}\mathcal{D}_{\mathbf{k}} = (\partial_{k_i}\mathcal{G}^\dagger_{\mathbf{k}})\Delta(\partial_{k_j}\mathcal{G}_{\mathbf{k}}) + (\partial_{k_j}\mathcal{G}^\dagger_{\mathbf{k}})\Delta(\partial_{k_i}\mathcal{G}_{\mathbf{k}}) - \left(\partial_{k_i}\partial_{k_j}\mathcal{G}^\dagger_{\mathbf{k}}\right)\Delta\mathcal{G}_{\mathbf{k}} - \mathcal{G}^\dagger_{\mathbf{k}}\Delta\left(\partial_{k_i}\partial_{k_j}\mathcal{G}_{\mathbf{k}}\right)\,. \tag{47}$$

The above expression simplifies if the diagonal matrix Δ is proportional to the identity, resulting in (Δ is now a scalar, not a matrix)

$$\partial_{q_i}\partial_{q_j}\mathcal{D}_{\mathbf{k}} = 2\Delta\left[\partial_{k_i}\mathcal{G}^\dagger_{\mathbf{k}}\partial_{k_j}\mathcal{G}_{\mathbf{k}} + \partial_{k_j}\mathcal{G}^\dagger_{\mathbf{k}}\partial_{k_i}\mathcal{G}_{\mathbf{k}}\right]\,. \tag{48}$$

In deriving the above equation we have used the identity $\partial_{k_i}\partial_{k_j}(\mathcal{G}^\dagger_{\mathbf{k}}\mathcal{G}_{\mathbf{k}}) = 0$, a consequence of the fact that $\mathcal{G}^\dagger_{\mathbf{k}}\mathcal{G}_{\mathbf{k}} = \mathbf{1}$. The component of the superfluid weight $D_{s,3}$ results from the combination of the second term in Eq. (39) and the last term in Eq. (40). It contains both a term which is possibly finite at zero temperature (see Eq. (14) in the main text) and a negative term which in the single-band case corresponds to the normal fluid component. $D_{{\rm s},3}$ must be present in order to preserve the invariance of the superfluid weight, an observable quantity, under gauge transformations. In this context the gauge transformations are related to an inherent ambiguity in the definition of the Bloch wavefunctions namely the possibility of applying a transformation of the form $\mathcal{G}_{\mathbf{k}} \to \mathcal{G}_{\mathbf{k}}\mathcal{A}_{\mathbf{k}}$ with $\mathcal{A}_{\mathbf{k}}$ a unitary matrix subject to the additional constrain

$$[\varepsilon_{\mathbf{k}}, \mathcal{A}_{\mathbf{k}}] = 0\,. \tag{49}$$

This ensures that the form of the BdG Hamiltonian $H_{\mathbf{k}}(\mathbf{q})$ in Eq. (6) in the main text is preserved under the transformation. If the band dispersions are nondegenerate then $\mathcal{A}_{\mathbf{k}}$ is simply a diagonal matrix of phases $\mathcal{A}_{\mathbf{k}} = \mathrm{diag}(e^{i\phi_n(\mathbf{k})})$. $D_{\mathrm{s},1}$ is invariant under such a transformation since one has $\mathcal{V}_{\mathbf{k}} \to \mathcal{A}^\dagger_{\mathbf{k}}\mathcal{V}_{\mathbf{k}}$ and Eq. (49) implies that $\mathcal{A}_{\mathbf{k}}$ commutes with the derivatives of $\varepsilon_{\mathbf{k}}$. This means that also the sum $D_{\mathrm{s},2} + D_{\mathrm{s},3}$ is gauge invariant. Below we show in a specific case that, by a suitable gauge transformation, it is possible to make the component $D_{\mathrm{s},3}$ vanish leaving only $D_{\mathrm{s},1}$ and $D_{\mathrm{s},2}$. But it is not clear if this is possible in general and it is convenient to allow some freedom in the choice of gauge. The calculation of the superfluid weight in the multiband case requires only the solution of the ground state problem, namely the quantities $\mathcal{U}_{\mathbf{k}}, \mathcal{V}_{\mathbf{k}}, E_{n\mathbf{k}}, \Delta_\alpha$, and the only additional ingredients with respect to the single-band case are the Bloch functions and their derivatives $\partial_{k_i}\mathcal{G}_{\mathbf{k}}$ and $\partial_{k_i}\partial_{k_j}\mathcal{G}_{\mathbf{k}}$ that enter in $D_{s,2}$ and $D_{s,3}$. It is easy to check that in the zero temperature limit Eqs. (41)-(46) reduce to the zero temperature result in the main text (Eqs. (12)-(14)). Eqs. (41)-(46) are very general and can be applied to a number of lattice models.

We also provide explicit expressions in terms of the matrices $\mathcal{U}_{\mathbf{k}}$ and $\mathcal{V}_{\mathbf{k}}$ and the quasiparticle energies $E_{n\mathbf{k}}$ of some correlation functions that are useful for the implementation of the self-consistency loop numerically or to check the self-consistency of an analytical solution. The first is the so-called anomalous Green function

$$F_{\mathbf{i}\alpha,\mathbf{j}\beta} = \langle \hat{c}_{\mathbf{i}\alpha\uparrow}\hat{c}_{\mathbf{j}\beta\downarrow}\rangle = \frac{1}{N_{\mathrm{c}}}\sum_{\mathbf{k}} e^{i\mathbf{k}\cdot(\mathbf{r}_{\mathbf{i}\alpha}-\mathbf{r}_{\mathbf{j}\beta})}\left[\mathcal{G}_{\mathbf{k}}\left(\mathcal{U}_{\mathbf{k}}\mathcal{V}^\dagger_{\mathbf{k}} - \mathcal{U}_{\mathbf{k}}\frac{1}{e^{\beta E^>_{\mathbf{k}}}+1}\mathcal{V}^\dagger_{\mathbf{k}} - \mathcal{V}_{\mathbf{k}}\frac{1}{e^{\beta E^>_{\mathbf{k}}}+1}\mathcal{U}^\dagger_{\mathbf{k}}\right)\mathcal{G}^\dagger_{\mathbf{k}}\right]_{\alpha,\beta}. \tag{50}$$

The unitary of $\mathcal{W}_{\mathbf{k}}(\mathbf{q})$ implies that $\mathcal{U}_{\mathbf{k}}\mathcal{V}^\dagger_{\mathbf{k}} = \mathcal{V}_{\mathbf{k}}\mathcal{U}^\dagger_{\mathbf{k}}$ which means that Eq. (50) is an Hermitian matrix $F_{\mathbf{i}\alpha,\mathbf{j}\beta} = F^*_{\mathbf{j}\beta,\mathbf{i}\alpha}$ and, therefore, the pairing potential $\Delta_\alpha = U\langle \hat{c}_{\mathbf{i}\alpha\uparrow}\hat{c}_{\mathbf{i}\alpha\downarrow}\rangle = UF_{\mathbf{i}\alpha,\mathbf{i}\alpha}$ is real. Another correlation function of interest is the usual Green function G

$$G_{\mathbf{i}\alpha,\mathbf{j}\beta} = \langle \hat{c}^\dagger_{\mathbf{j}\beta\uparrow}\hat{c}_{\mathbf{i}\alpha\uparrow}\rangle = \langle \hat{c}^\dagger_{\mathbf{i}\alpha\downarrow}\hat{c}_{\mathbf{j}\beta\downarrow}\rangle = \frac{1}{N_{\mathrm{c}}}\sum_{\mathbf{k}} e^{-i\mathbf{k}\cdot(\mathbf{r}_{\mathbf{i}\alpha}-\mathbf{r}_{\mathbf{j}\beta})}\left[\mathcal{G}_{\mathbf{k}}\left(\mathcal{U}_{\mathbf{k}}\frac{1}{e^{\beta E^>_{\mathbf{k}}}+1}\mathcal{U}^\dagger_{\mathbf{k}} + \mathcal{V}_{\mathbf{k}}\frac{1}{e^{-\beta E^>_{\mathbf{k}}}+1}\mathcal{V}^\dagger_{\mathbf{k}}\right)\mathcal{G}^\dagger_{\mathbf{k}}\right]_{\alpha,\beta}. \tag{51}$$

The total particle density is immediately obtained from the Green's function $n_{\mathbf{i}\alpha} = \langle \hat{c}^\dagger_{\mathbf{i}\alpha\uparrow}\hat{c}_{\mathbf{i}\alpha\uparrow}\rangle + \langle \hat{c}^\dagger_{\mathbf{i}\alpha\downarrow}\hat{c}_{\mathbf{i}\alpha\downarrow}\rangle = 2G_{\mathbf{i}\alpha,\mathbf{i}\alpha}$.

Supplementary Note 4: Superfluid density of the time-reversal invariant Harper-Hubbard model

We now specialize our result for the superfluid density in a generic multiband system to the case of the Harper model. The band dispersions $\varepsilon_{n\mathbf{k}}$ and periodic Bloch functions $g_{n\mathbf{k}}$ of the Harper model with commensurate flux per plaquette $n_\phi = 1/Q = 1/N_{\mathrm{orb}}$ are given by the Harper equation

$$e^{ik_ya}g_{n\mathbf{k}}(\alpha+1) + e^{-ik_ya}g_{n\mathbf{k}}(\alpha-1) + 2\cos\left(k_xa - 2\pi\frac{\alpha}{Q}\right)g_{n\mathbf{k}}(\alpha) = -\frac{\varepsilon_{n\mathbf{k}}}{J}g_{n\mathbf{k}}(\alpha)\,, \tag{52}$$

with periodic boundary conditions $g_{n\mathbf{k}}(\alpha) = g_{n\mathbf{k}}(\alpha+Q)$. A useful property of the Bloch functions that can be derived from the definition is

$$\varepsilon_{n\left(\mathbf{k}+\frac{2\pi}{Qa}\hat{\mathbf{k}}_x\right)} = \varepsilon_{n\mathbf{k}}, \quad g_{n\left(\mathbf{k}+\frac{2\pi}{Qa}\hat{\mathbf{k}}_x\right)}(\alpha) = g_{n\mathbf{k}}(\alpha-1) \qquad \text{with} \quad \hat{\mathbf{k}}_x = \begin{pmatrix}1\\0\end{pmatrix}. \tag{53}$$

By means of the above identity it is shown below that a constant gap function $\Delta_\alpha = \Delta$ is a self consistent solution of the gap equation, therefore in the following Δ is a positive real number and not a matrix as defined previously. This implies that $\mathcal{G}^\dagger_{\mathbf{k}}\Delta\mathcal{G}_{\mathbf{k}} = \Delta\mathcal{G}^\dagger_{\mathbf{k}}\mathcal{G}_{\mathbf{k}} = \Delta\mathbf{1}$. In this case the BdG Hamiltonian $H_{\mathbf{k}}(\mathbf{q}=0)$

can be easily diagonalized and the solution reads

$$[\mathcal{U}_{\mathbf{k}}]_{n,n'} = u_{n\mathbf{k}}\delta_{n,n'} \quad \text{with} \quad u_{n\mathbf{k}} = \frac{1}{\sqrt{2}}\left(1 + \frac{\varepsilon_{n\mathbf{k}} - \mu}{E_{n\mathbf{k}}}\right)^{\frac{1}{2}}, \tag{54}$$

$$[\mathcal{V}_{\mathbf{k}}]_{n,n'} = v_{n\mathbf{k}}\delta_{n,n'} \quad \text{with} \quad v_{n\mathbf{k}} = \frac{1}{\sqrt{2}}\left(1 - \frac{\varepsilon_{n\mathbf{k}} - \mu}{E_{n\mathbf{k}}}\right)^{\frac{1}{2}}, \tag{55}$$

$$E_{n\mathbf{k}} = \sqrt{(\varepsilon_{n\mathbf{k}} - \mu)^2 + \Delta^2}\,. \tag{56}$$

A fundamental object for what follows is the projector operator $P_{n\mathbf{k}}$ defined in terms of its matrix elements

$$[P_{n\mathbf{k}}]_{\alpha,\beta} = \left[\mathcal{G}_{\mathbf{k}}\right]_{\alpha,n}\left[\mathcal{G}^{\dagger}_{\mathbf{k}}\right]_{n,\beta}. \tag{57}$$

This is a projection operator since it is positive $P_{n\mathbf{k}} > 0$ and idempotent $P^2_{n\mathbf{k}} = P_{n\mathbf{k}}$. The above definition is immediately generalized to the projection operator onto a given set of bands simply by summing over the corresponding subset of values of n. Note that Eq. (53) imply that

$$\sum_m \left[P_{n\left(\mathbf{k}+\frac{2\pi m}{Qa}\hat{\mathbf{k}}_x\right)}\right]_{\alpha,\alpha} = \sum_m g_{n\left(\mathbf{k}+\frac{2\pi m}{Qa}\hat{\mathbf{k}}_x\right)}(\alpha) g^*_{n\left(\mathbf{k}+\frac{2\pi m}{Qa}\hat{\mathbf{k}}_x\right)}(\alpha) = \sum_m |g_{n\mathbf{k}}(\alpha - m)|^2 = 1\,. \tag{58}$$

Thus the gap equation at zero temperature reads

$$\begin{aligned}\Delta = \Delta_\alpha = UF_{\mathbf{i}\alpha,\mathbf{i}\alpha} &= \frac{U}{N_{\mathrm{c}}}\sum_{\mathbf{k},n} u_{n\mathbf{k}}v_{n\mathbf{k}}\tanh\frac{\beta E_{n\mathbf{k}}}{2}[P_{n\mathbf{k}}]_{\alpha,\alpha} \\ &= \frac{U}{N_{\mathrm{c}}}\sum_n \sum_{\mathbf{k}\in\,\text{red.B.Z.}} \frac{\Delta}{2E_{n\mathbf{k}}}\tanh\frac{\beta E_{n\mathbf{k}}}{2}\sum_m \left[P_{n\left(\mathbf{k}+\frac{2\pi m}{Qa}\hat{\mathbf{k}}_x\right)}\right]_{\alpha,\alpha} \\ &= \frac{U}{N_{\mathrm{c}}}\sum_n \sum_{\mathbf{k}\in\,\text{red.B.Z.}} \frac{\Delta}{2E_{n\mathbf{k}}}\tanh\frac{\beta E_{n\mathbf{k}}}{2}\,,\end{aligned} \tag{59}$$

In the second and third line of the above equation the vector $\mathbf{k}$ takes values on the reduced Brillouin zone $k_x, k_y \in [-\pi/(Qa), \pi/(Qa)]$ since the degeneracy due to Eq. (53) has been taken care of by the summation over m in the second line. Analogously the average number of particles per lattice site $n_{\mathrm{p}} = n_{\mathbf{i}\alpha}$ is fixed by the equation

$$\frac{n_{\mathrm{p}}}{2} = \frac{1}{N_c}\sum_n \sum_{\mathbf{k}\in\,\text{red.B.Z.}} \frac{1}{2}\left(1 - \frac{\varepsilon_{n\mathbf{k}} - \mu}{E_{n\mathbf{k}}}\tanh\frac{\beta E_{n\mathbf{k}}}{2}\right). \tag{60}$$

If a solution of the coupled equations (59)-(60) is found then our initial choice of a constant pairing potential is self-consistent and this is in general the case since we have two equations with two unknowns.

Up to now all of our calculations have not involved any approximations other than the mean-field one. We now use some approximate results for the eigenfunctions and eigenvalues of the Harper model as detailed in Ref. 5. It has been shown that for $Q \gg 1$ the first lowest laying bands are separated by a gap which is an algebraic function of Q, while the bandwidth of each band is exponentially suppressed ($\max_{\mathbf{k}}\varepsilon_{n\mathbf{k}} - \min_{\mathbf{k}}\varepsilon_{n\mathbf{k}} \propto e^{-\alpha Q}$ with $\alpha \approx 1$). This corresponds to the the fact that the Harper model bands approach the perfectly flat Landau levels in the continuum limit. We call $\varepsilon_{\bar{n}} \approx \varepsilon_{\bar{n}\mathbf{k}}$ the average energy of the $\bar{n}$-th band and choose a value of the interaction strength U such that Eq. (15) in the main text is satisfied, meaning that interband effects and effects due to a nonflat band dispersion are neglected. As a consequence the summation over the wavevector $\mathbf{k}$ produces $N_{\mathrm{c}}/Q = N_{\mathrm{c}}n_\phi$ identical terms in Eq. (59)-(60). We write $n_{\mathrm{p}}/(2n_\phi) = \bar{n} + \nu$ with $\bar{n}$ the integer number of the highest (partially or completely) occupied band of the Harper model and with $0 < \nu < 1$ the filling of factor of the same band. The filling factor ν is spin resolved and is the same for up and down spins. The bands are numbered starting from $n = 0$ up to $n = Q - 1$ to conform with the usual Landau level notation. In the flat band approximation $\varepsilon_{\bar{n}\mathbf{k}} \approx \varepsilon_{\bar{n}}$, one

has from Eq. (56) that $E_{\bar{n}\mathbf{k}} \approx E_{\bar{n}}$ and since we expect $U \sim E_{\bar{n}} \ll E_{n\mathbf{k}}$ for $n \neq \bar{n}$, only the $n = \bar{n}$ term is relevant in Eq. (59). By cancelling Δ on both sides one obtains the equation for $E_{\bar{n}}$

$$E_{\bar{n}} = \frac{Un_\phi}{2} \tanh \frac{\beta E_{\bar{n}}}{2} \,. \tag{61}$$

This equation has nonzero solution only for $\beta U n_\phi/4 > 1$. Indeed $k_{\rm B}T_{\rm c} = Un_\phi/4$ is the mean-field critical temperature at half filling (see below). Close to the critical temperature it is possible to use the approximation $\tanh x \approx x - \frac{x^3}{3}$ and derive $E_{\bar{n}}(T \lesssim T_{\rm c}) \approx 2\sqrt{3}k_BT\sqrt{1-T/T_{\rm c}}$. Using Eq. (60) as well it is easy to obtain the approximate self consistent solution at finite temperature

$$E_{n\mathbf{k}} = \begin{cases} E_{\bar{n}}, & n = \bar{n} \\ |\varepsilon_{n\mathbf{k}} - \mu|, & n \neq \bar{n}\,, \end{cases} \tag{62}$$

$$\mu = \varepsilon_{\bar{n}} + Un_\phi\left(\nu - \frac{1}{2}\right)\,, \tag{63}$$

$$\Delta = \frac{Un_\phi}{2}\sqrt{\left(\frac{2E_{\bar{n}}}{Un_\phi}\right)^2 - (2\nu-1)^2}\,. \tag{64}$$

If the argument in the square root of Eq. (64) is negative then $\Delta = 0$ even if Eq. (61) has a solution. This can happen away from half-filling. Instead at half filling $\Delta = E_{\bar{n}}$. The zero temperature limit of the above solution is given by Eq. (16)-(19) in the main text. Since the Harper model has been recently implemented in ultracold gases we test the validity of the isolated flat-band approximation (Eqs. (61)-(64)) against the full self-consistent solution of Eq. (59)-(60) in Supplementary Figure 2. For reasonable (not too small values) of n_ϕ and U the flat-band limit is indeed realized to a good approximation.

In the derivation of the approximate self-consistent solution (61)-(64) we have used the property (53) specific of the Harper model. However it is generally valid in the case where $\Delta_\alpha = \Delta$ with Δ a real scalar. Indeed simply using this assumptions, which leads to Eqs. (54)-(56), and the condition (15) in the main text one obtains the two coupled self-consistency equations

$$\Delta = \Delta_\alpha = U\frac{\Delta}{2E_{\bar{n}}}\tanh\left(\frac{\beta E_{\bar{n}}}{2}\right)\left(\frac{1}{N_{\rm c}}\sum_{\mathbf{k}}[P_{\bar{n}\mathbf{k}}]_{\alpha,\alpha}\right)\,, \tag{65}$$

$$\frac{n_{\rm p}}{2} = \frac{n_{\mathbf{i}\alpha}}{2} = \left[\bar{n} + \frac{1}{2}\left(1 - \frac{\varepsilon_{\bar{n}} - \mu}{E_{\bar{n}}}\right)\tanh\left(\frac{\beta E_{\bar{n}}}{2}\right)\right]\left(\frac{1}{N_{\rm c}}\sum_{\mathbf{k}}[P_{\bar{n}\mathbf{k}}]_{\alpha,\alpha}\right)\,. \tag{66}$$

The projector on a single band is normalized according to $\mathrm{Tr}\,P_{\bar{n}\mathbf{k}} = 1$ and this implies $N_{\rm c}^{-1}\sum_{\mathbf{k}}[P_{\bar{n}\mathbf{k}}]_{\alpha,\alpha} = N_{\rm orb}^{-1}$. The result in Eqs. (61)-(64) is thus recovered with $n_\phi = N_{\rm orb}^{-1}$.

We are now in position to evaluate the various contributions to the superfluid weight. The diagonal superfluid weight is zero in the flat-band approximation. The other contributions can be evaluated straightforwardly and the result is

$$[D_{\rm s}]_{i,j} = \frac{2}{\pi\hbar^2}\frac{\Delta^2(T)}{Un_\phi}\mathcal{M}^{\rm R}_{ij}\,, \tag{67}$$

where the temperature enters only in the energy gap $\Delta(T)$. This result reduces to Eq. (20) in the main text at zero temperature. In deriving the above equation we have used the fact that $u_{n\mathbf{k}}v_{n\mathbf{k}} \approx 0$ for $n \neq \bar{n}$. We have also neglected terms with $n \neq \bar{n}$ in Eq. (46) since $E_{\bar{n}} \ll E_{n\neq\bar{n}}$ according to Eq. (62). The matrix $\mathcal{M}^{\rm R}_{ij} = \mathrm{Re}(\mathcal{M}_{ij})$ is the real part of a Hermitian matrix defined as

$$\mathcal{M}_{ij} = \frac{2\pi}{A}\sum_{\mathbf{k}}\mathcal{B}_{ij}(\mathbf{k}) = \frac{1}{2\pi}\int_{\rm B.Z.} d^2\mathbf{k}\,\mathcal{B}_{ij}(\mathbf{k}) = \frac{1}{2\pi}\int_{\rm B.Z.} d^2\mathbf{k}\,2\left(\mathrm{Tr}\left[(\partial_{k_i}\bar{\mathcal{G}}^\dagger_{\mathbf{k}})(\partial_{k_j}\bar{\mathcal{G}}_{\mathbf{k}})\right] + \mathrm{Tr}\left[\bar{\mathcal{G}}^\dagger_{\mathbf{k}}(\partial_{k_i}\bar{\mathcal{G}}_{\mathbf{k}})\bar{\mathcal{G}}^\dagger_{\mathbf{k}}(\partial_{k_j}\bar{\mathcal{G}}_{\mathbf{k}})\right]\right)\,. \tag{68}$$

The integration is over the full Brillouin zone. The first term in parenthesis in the above equation corresponds to $D_{\mathrm{s},2}$ while the other one corresponds to $D_{\mathrm{s},3}$. Crucially the matrix $\mathcal{M}$ is defined in terms of the matrix $\bar{\mathcal{G}}_{\mathbf{k}}$ which is now a $Q \times 1$ column vector (in general a rectangular matrix), defined as the projection of $\mathcal{G}_{\mathbf{k}}$ on the Bloch wavefunctions corresponding to the $\bar{n}$-th band

$$[\bar{\mathcal{G}}_{\mathbf{k}}]_{\alpha,1} = [\mathcal{G}_{\mathbf{k}}]_{\alpha,\bar{n}}\,, \quad \bar{\mathcal{G}}^\dagger_{\mathbf{k}}\bar{\mathcal{G}}_{\mathbf{k}} = \mathbf{1}\,, \quad \bar{\mathcal{G}}_{\mathbf{k}}\bar{\mathcal{G}}^\dagger_{\mathbf{k}} = P_{\bar{n}\mathbf{k}}\,. \tag{69}$$

It can be easily shown that if $\bar{\mathcal{G}}_{\mathbf{k}}$ is a square unitary then $\mathcal{M}$ is zero. In our context in fact a square unitary matrix is a pure gauge transformation which must not have observable consequences. The matrix $\mathcal{M}$ is also positive semidefinite $\mathcal{M} \geq 0$ since it can be written in the form

$$\mathcal{M}_{ij} = \frac{1}{2\pi}\int_{\mathrm{B.Z.}} d^2\mathbf{k}\, 2\mathrm{Tr}\left[(\partial_{k_i}\bar{\mathcal{G}}^\dagger_{\mathbf{k}})(\mathbf{1} - P_{\bar{n}\mathbf{k}})(\partial_{k_j}\bar{\mathcal{G}}_{\mathbf{k}})\right]\,. \tag{70}$$

The invariant quantity $\mathcal{B}_{ij}(\mathbf{k})$ is called the quantum geometric tensor, its real part is the quantum metric, while the imaginary part of $\mathcal{B}_{ij}(\mathbf{k})$ is the well-known Berry curvature, and its integral over the Brillouin zone is the Chern number $\mathrm{Im}(\mathcal{M}_{ij}) = \mathcal{M}^{\mathrm{I}}_{ij} = \epsilon_{ij}C$, generalized to a set of bands (a composite band)[4] ($\epsilon_{ij} = -\epsilon_{ji}$ is the Levi-Civita symbol). The second term in parenthesis in Eq. (68) is real and does not show up in the Berry curvature. In can be easily verified that the quantum geometric tensor is an invariant under gauge transformations $\bar{\mathcal{G}}_{\mathbf{k}} \to \bar{\mathcal{G}}_{\mathbf{k}}\mathcal{A}_{\mathbf{k}}$ where $\mathcal{A}_{\mathbf{k}}$ is a unitary matrix with dimension equal to the number of columns of $\bar{\mathcal{G}}_{\mathbf{k}}$.

We now want to evaluate $\mathcal{M}_{ij}$ using a suitable approximation for the wavefunctions of the Harper model. Such an approximation can be obtained by taking the Harper equation (52) and replacing the cosine potential with its second order expansion around the minimum and furthermore replacing the discrete differences with derivatives. We obtain the equation of an ordinary harmonic oscillator for the Bloch wavefunction $\psi(\mathbf{r}_{\mathbf{i}\alpha}) = e^{ik_x i_x a}\psi(\alpha + Qi_y)$

$$-\frac{1}{2}\psi''(\alpha) + \frac{1}{2}\omega^2\left(\alpha - \frac{Qk_xa}{2\pi}\right)^2\psi(\alpha) = \frac{1}{2}\left(\frac{\varepsilon}{J} + 4\right)\psi(\alpha) \quad \text{with} \quad \omega = \frac{2\pi}{Q}\,. \tag{71}$$

The variable α is now a continuous variable. We are neglecting effects due to the tunneling between the minima of the cosine potential in the Harper equation (52) in the main text. These corrections can be shown to be of the same order of the exponentially small bandwidth[5] which we neglect in our treatment. The above equation is precisely the equation that one obtains when solving the problem of a quantum mechanical particle in a magnetic field introduced by means of the Landau gauge (note the shift of the harmonic potential proportional to k_x). The solutions are the harmonic oscillator wavefunctions $\varphi_n(x)$ (Gaussian function times an Hermite polynomial) which corresponds to the different Landau levels. It is also useful to introduce annihilation and creation operators to manipulate such functions ($\hat{x} = x$ and $\hat{p} = -i\partial_x$)

$$\hat{a} = \sqrt{\frac{\omega}{2}}\hat{x} + \frac{i}{\sqrt{2\omega}}\hat{p}, \quad \hat{a}\varphi_n = \sqrt{n}\varphi_{n-1}\,, \tag{72}$$

$$\hat{a}^\dagger = \sqrt{\frac{\omega}{2}}\hat{x} - \frac{i}{\sqrt{2\omega}}\hat{p}, \quad \hat{a}^\dagger\varphi_n = \sqrt{n+1}\varphi_{n+1}\,. \tag{73}$$

Eq. (71) can be rewritten in terms of the annihilation and creation operators in the form

$$\omega\left(\hat{a}^\dagger\hat{a} + \frac{1}{2}\right)\psi(\alpha) = \frac{1}{2}\left(\frac{\varepsilon}{J} + 4\right)\psi(\alpha)\,. \tag{74}$$

The lattice periodicity is taken into account by imposing that the Bloch wavefunction transform in the proper way under translations, namely one has

$$\psi_{n\mathbf{k}}(\mathbf{r}_{\mathbf{i}\alpha}) = \frac{1}{\sqrt{N_c}}\sum_s e^{i(k_x i_x + Qk_y s)a}\varphi_n\left(\alpha + Qi_y - Qs - \frac{Qk_xa}{2\pi}\right)\,. \tag{75}$$

The above wavefunctions satisfy the properties required by Bloch theorem, namely $\psi_{n\mathbf{k}}(\mathbf{r}_{\mathbf{i}\alpha} + a\hat{\mathbf{x}}) = e^{ik_x a}\psi_{n\mathbf{k}}(\mathbf{r}_{\mathbf{i}\alpha})$ and $\psi_{n\mathbf{k}}(\mathbf{r}_{\mathbf{i}\alpha} + aQ\hat{\mathbf{y}}) = e^{iQk_y a}\psi_{n\mathbf{k}}(\mathbf{r}_{\mathbf{i}\alpha})$. In order to ease the notation in the following we label the positions of the lattice sites in the square lattice in the more natural way $\mathbf{r}_{\mathbf{i}} = a(i_x\hat{\mathbf{x}} + i_y\hat{\mathbf{y}})$, rather than $\mathbf{r}_{\mathbf{i}\alpha} = a(i_x\hat{\mathbf{x}} + (\alpha + Qi_y)\hat{\mathbf{y}})$. Checking that the above wavefunctions are orthogonal and normalized is a good way to get a better feeling of the approximations that have been made (the number of lattice sites is $N_{\rm s} = L^2$ with $L = QM$ and the therefore then number of unit cells is $N_{\rm c} = N_{\rm s}/Q = QM^2$)

$$\begin{aligned}
\sum_{\mathbf{j}} \psi^*_{n\mathbf{k}'}(\mathbf{r}_{\mathbf{j}})\psi_{n\mathbf{k}}(\mathbf{r}_{\mathbf{j}}) &= \frac{1}{QM^2}\sum_{s,s'}\sum_{j_x,j_y} e^{i(k_x-k'_x)j_x a} e^{iQ(k_y s - k'_y s')a} \times \\
&\times \varphi_n\left(j_y - Qs - \frac{Qk_x a}{2\pi}\right)\varphi_n\left(j_y - Qs' - \frac{Qk'_x a}{2\pi}\right) \\
&= \frac{\delta_{k_x,k'_x}}{M}\sum_{s,s'}\sum_{j_y} e^{iQ(k_y s - k'_y s')a}\varphi_n\left(j_y - Qs - \frac{Qk_x a}{2\pi}\right)\varphi_n\left(j_y - Qs' - \frac{Qk_x a}{2\pi}\right) \\
&= \frac{\delta_{k_x,k'_x}}{M}\sum_{s}\sum_{j_y} e^{iQ(k_y - k'_y)sa}\varphi^2_n\left(j_y - Qs - \frac{Qk_x a}{2\pi}\right) = \frac{\delta_{k_x,k'_x}}{M}\sum_{s} e^{iQ(k_y-k'_y)sa} \\
&= \delta_{k_x,k'_x}\delta_{k_y,k'_y}\,.
\end{aligned} \tag{76}$$

We have neglected terms where $s \neq s'$ since the harmonic oscillator wavefunctions are well localized inside the large (Q sites) magnetic unit cell and their overlap with wavefunctions in neighbouring magnetic unit cell is exponentially small. Moreover we approximate the sum over j_y with the integral $\sum_{j_y}\varphi^2_n(j_y) \approx \int dx\,\varphi^2_n(x) = 1$, which leads to an error of the same order as it can be checked with the Euler-MacLaurin formula. The harmonic oscillator wavefunctions are normalized in the usual way. The *periodic* Bloch functions $g_{n\mathbf{k}}(j_y)$ are thus (the same as Eq. (25) in the main text with a different labelling)

$$g_{n\mathbf{k}}(j_y) = \sum_s e^{-ik_y(j_y - Qs)a}\varphi_n\left(j_y - Qs - \frac{Qk_x a}{2\pi}\right)\,. \tag{77}$$

We can now calculate $\mathcal{B}_{ij}(\mathbf{k})$ within this approximation for the periodic Bloch functions. We consider first the quantity $\bar{\mathcal{G}}^\dagger_{\mathbf{k}}(\partial_{k_j}\bar{\mathcal{G}}_{\mathbf{k}})$ that enters the second term in parenthesis in Eq. (68)

$$\begin{aligned}
\bar{\mathcal{G}}^\dagger_{\mathbf{k}}(\partial_{k_x}\bar{\mathcal{G}}_{\mathbf{k}}) &= \sum_{j_y=1}^{Q} g^*_{\bar{n}\mathbf{k}}(j_y)\partial_{k_x} g_{\bar{n}\mathbf{k}}(j_y) \\
&= \sum_{j_y=1}^{Q}\sum_{s,s'} e^{-iQ(s-s')k_y}\varphi_{\bar{n}}\left(j_y - Qs - \frac{Qk_x a}{2\pi}\right)\partial_{k_x}\varphi_{\bar{n}}\left(j_y - Qs' - \frac{Qk_x a}{2\pi}\right) \\
&= \sum_{j_y=1}^{Q}\sum_{s}\varphi_{\bar{n}}\left(j_y - Qs - \frac{Qk_x a}{2\pi}\right)\partial_{k_x}\varphi_{\bar{n}}\left(j_y - Qs - \frac{Qk_x a}{2\pi}\right) \\
&= -\frac{Qa}{2\pi}\sum_{j_y}\varphi_{\bar{n}}\left(j_y\right)\sqrt{\frac{\omega}{2}}(\hat{a} - \hat{a}^\dagger)\varphi_{\bar{n}}(j_y) \\
&= -\frac{Qa}{2\pi}\sqrt{\frac{\omega}{2}}\sum_{j_y}\varphi_{\bar{n}}\left(j_y\right)\left(\sqrt{\bar{n}}\varphi_{\bar{n}-1}(j_y) - \sqrt{\bar{n}+1}\varphi_{\bar{n}+1}(j_y)\right) = 0\,.
\end{aligned} \tag{78}$$

We have combined the sum over $j_y = 1, \ldots, Q$ and the sum over s in a single sum over all integer j_y. This sum over j_y has then been approximated by an integral and the overlap of orthogonal functions φ_n carried out as usual leading to a zero result. One can check that the choice of the periodic Bloch functions in Eq. (77) leads to $\bar{\mathcal{G}}^\dagger_{\mathbf{k}}(\partial_{k_y}\bar{\mathcal{G}}_{\mathbf{k}}) \neq 0$. However we can perform a gauge transformation of the

form $g_{n\mathbf{k}} \to e^{iQk_xk_ya^2/(2\pi)}g_{n\mathbf{k}}$. Going through the calculation along the same line of Eq. (78) one obtains something proportional to $\sum_{j_y} j_y\varphi_{\bar{n}}^2(j_y) \approx \int dx\, x\varphi_{\bar{n}}^2(x) = 0$. We will make use of this gauge choice when convenient.

First we calculate $\mathcal{B}_{xy}(\mathbf{k})$. We only need to evaluate $\mathrm{Tr}\left[(\partial_{k_x}\bar{\mathcal{G}}_{\mathbf{k}}^\dagger)(\partial_{k_y}\bar{\mathcal{G}}_{\mathbf{k}})\right]$ since the second term in parenthesis in Eq. (68) is zero according to Eq. (78)

$$\begin{aligned}
\frac{1}{2}\mathcal{B}_{xy}(\mathbf{k}) &= \mathrm{Tr}\left[(\partial_{k_x}\bar{\mathcal{G}}_{\mathbf{k}}^\dagger)(\partial_{k_y}\bar{\mathcal{G}}_{\mathbf{k}})\right] = \sum_{j_y=1}^{Q} \partial_{k_x} g_{\bar{n}\mathbf{k}}^*(j_y)\partial_{k_y} g_{\bar{n}\mathbf{k}}(j_y) \\
&= \sum_{j_y=1}^{Q}\sum_{s,s'} \partial_{k_x}\left[e^{ik_y(j_y-Qs)a}\varphi_{\bar{n}}\left(j_y - Qs - \frac{Qk_xa}{2\pi}\right)\right]\partial_{k_y}\left[e^{-ik_y(j_y-Qs')a}\varphi_{\bar{n}}\left(j_y - Qs' - \frac{Qk_xa}{2\pi}\right)\right] \\
&= \sum_{j_y} \partial_{k_x}\left[e^{ik_yj_ya}\varphi_{\bar{n}}\left(j_y - \frac{Qk_xa}{2\pi}\right)\right]\partial_{k_y}\left[e^{-ik_yj_ya}\varphi_{\bar{n}}\left(j_y - \frac{Qk_xa}{2\pi}\right)\right] \\
&= \frac{iQa^2}{2\pi}\sum_{j_y}\varphi_{\bar{n}}'\left(j_y - \frac{Qk_xa}{2\pi}\right)j_y\varphi_{\bar{n}}\left(j_y - \frac{Qk_xa}{2\pi}\right) \\
&= \frac{iQa^2}{2\pi}\sum_{j_y}\left[\sqrt{\frac{\omega}{2}}\left(\hat{a} - \hat{a}^\dagger\right)\varphi_{\bar{n}}\left(j_y - \frac{Qk_xa}{2\pi}\right)\right]\left[\left(\frac{1}{\sqrt{2\omega}}\left(\hat{a} + \hat{a}^\dagger\right) + \frac{Qk_xa}{2\pi}\right)\varphi_{\bar{n}}\left(j_y - \frac{Qk_xa}{2\pi}\right)\right] \\
&= \frac{iQa^2}{4\pi}\sum_{j_y}\left[\sqrt{\bar{n}}\varphi_{\bar{n}-1}(j_y) - \sqrt{\bar{n}+1}\varphi_{\bar{n}+1}(j_y)\right]\left[\sqrt{\bar{n}}\varphi_{\bar{n}-1}(j_y) + \sqrt{\bar{n}+1}\varphi_{\bar{n}+1}(j_y)\right] = -\frac{iQa^2}{4\pi}\,.
\end{aligned} \tag{79}$$

Next we evaluate $\mathcal{B}_{xx}(\mathbf{k})$. Again we need only need to evaluate $\mathrm{Tr}\left[(\partial_{k_x}\bar{\mathcal{G}}_{\mathbf{k}}^\dagger)(\partial_{k_x}\bar{\mathcal{G}}_{\mathbf{k}})\right]$ according to Eq. (78)

$$\begin{aligned}
\frac{1}{2}\mathcal{B}_{xx}(\mathbf{k}) &= \mathrm{Tr}\left[(\partial_{k_x}\bar{\mathcal{G}}_{\mathbf{k}}^\dagger)(\partial_{k_x}\bar{\mathcal{G}}_{\mathbf{k}})\right] = \sum_{j_y=1}^{Q} \partial_{k_x} g_{\bar{n}\mathbf{k}}^*(j_y)\partial_{k_x} g_{\bar{n}\mathbf{k}}(j_y) \\
&= \sum_{j_y}\partial_{k_x}\varphi_{\bar{n}}\left(j_y - \frac{Qk_xa}{2\pi}\right)\partial_{k_x}\varphi_{\bar{n}}\left(j_y - \frac{Qk_xa}{2\pi}\right) \\
&= \left(\frac{Qa}{2\pi}\right)^2\sum_{j_y}\left[\sqrt{\frac{\omega}{2}}\left(\hat{a} - \hat{a}^\dagger\right)\varphi_{\bar{n}}\left(j_y\right)\right]\left[\sqrt{\frac{\omega}{2}}\left(\hat{a} - \hat{a}^\dagger\right)\varphi_{\bar{n}}\left(j_y\right)\right] \\
&= \frac{Qa^2}{4\pi}\sum_{j_y}\left[\sqrt{\bar{n}}\varphi_{\bar{n}-1}(j_y) - \sqrt{\bar{n}+1}\varphi_{\bar{n}+1}(j_y)\right]\left[\sqrt{\bar{n}}\varphi_{\bar{n}-1}(j_y) - \sqrt{\bar{n}+1}\varphi_{\bar{n}+1}(j_y)\right] \\
&= \frac{Qa^2}{4\pi}(2\bar{n}+1)\,.
\end{aligned} \tag{80}$$

For $\mathcal{B}_{yy}(\mathbf{k})$ we choose the modified gauge that ensures the vanishing of the second term in parenthesis in

Eq. (68)

$$
\begin{aligned}
\frac{1}{2}\mathcal{B}_{yy}(\mathbf{k}) &= \mathrm{Tr}\left[(\partial_{k_y}\bar{\mathcal{G}}^\dagger_{\mathbf{k}})(\partial_{k_y}\bar{\mathcal{G}}_{\mathbf{k}})\right] = \sum_{j_y=1}^{Q}\partial_{k_y}g^*_{\bar{n}\mathbf{k}}(j_y)\partial_{k_y}g_{\bar{n}\mathbf{k}}(j_y)\\
&= \sum_{j_y}\partial_{k_y}\left[e^{ik_y\left(j_y-\frac{Qk_xa}{2\pi}\right)a}\varphi_{\bar{n}}\left(j_y-\frac{Qk_xa}{2\pi}\right)\right]\partial_{k_y}\left[e^{-ik_y\left(j_y-\frac{Qk_xa}{2\pi}\right)a}\varphi_{\bar{n}}\left(j_y-\frac{Qk_xa}{2\pi}\right)\right]\\
&= a^2\sum_{j_y}\left[\left(j_y-\frac{Qk_xa}{2\pi}\right)\varphi_{\bar{n}}\left(j_y-\frac{Qk_xa}{2\pi}\right)\right]\left[\left(j_y-\frac{Qk_xa}{2\pi}\right)\varphi_{\bar{n}}\left(j_y-\frac{Qk_xa}{2\pi}\right)\right]\\
&= a^2\sum_{j_y}\left[\frac{1}{\sqrt{2\omega}}\left(\hat{a}+\hat{a}^\dagger\right)\varphi_{\bar{n}}\left(j_y\right)\right]\left[\frac{1}{\sqrt{2\omega}}\left(\hat{a}+\hat{a}^\dagger\right)\varphi_{\bar{n}}\left(j_y\right)\right]\\
&= \frac{Qa^2}{4\pi}\sum_{j_y}\left[\sqrt{\bar{n}}\varphi_{\bar{n}-1}(j_y)+\sqrt{\bar{n}+1}\varphi_{\bar{n}+1}(j_y)\right]\left[\sqrt{\bar{n}}\varphi_{\bar{n}-1}(j_y)+\sqrt{\bar{n}+1}\varphi_{\bar{n}+1}(j_y)\right]\\
&= \frac{Qa^2}{4\pi}(2\bar{n}+1)\,.
\end{aligned}
\tag{81}
$$

The quantum geometric tensor $\mathcal{B}_{ij}$ is constant over the Brillouin zone and the calculation of $\mathcal{M}$ is trivial (the integration is over the full Brillouin zone $k_x = [-\pi/a, \pi/a]$, $k_x = [-\pi/(Qa), \pi/(Qa)]$)

$$
\mathcal{M} = \frac{1}{2\pi}\int d^2\mathbf{k}\,\mathcal{B}(\mathbf{k}) = \frac{Qa^2}{(2\pi)^2}\int_{\mathrm{B.Z.}}d\mathbf{k}\begin{pmatrix}2\bar{n}+1 & -i\\ i & 2\bar{n}+1\end{pmatrix} = \begin{pmatrix}2\bar{n}+1 & -i\\ i & 2\bar{n}+1\end{pmatrix}\,. \tag{82}
$$

We obtain the result that for a Landau level the Chern number is $|C| = 1$ and that the superfluid density in the $\bar{n}$-th Landau level is proportional to $2\bar{n}+1$. In particular for the lowest Landau level the bound in Eq. (23) in the main text is saturated.

Supplementary Note 5: Bound on the localization functional for Wannier functions

Marzari and Vanderbilt consider the following functional that can be minimized numerically in the matrix $U_{\mathbf{k}}$ in Eq. (29) in order to construct maximally localized Wannier functions[6] (we consider the 2D case for simplicity)

$$
F = \sum_{\alpha}\left[\langle\mathbf{0}\alpha|\,r^2\,|\mathbf{0}\alpha\rangle - |\langle\mathbf{0}\alpha|\,\mathbf{r}\,|\mathbf{0}\alpha\rangle|^2\right]\,, \tag{83}
$$

$$
\langle\mathbf{0}\alpha|\,r^2\,|\mathbf{0}\alpha\rangle = \int_\Omega d^2\mathbf{r}\,|w_\alpha(\mathbf{r})|^2r^2 = \frac{A_\Omega}{(2\pi)^2}\int_{B.Z.}d^2\mathbf{k}\,\langle\boldsymbol{\nabla}_{\mathbf{k}}g_{\alpha\mathbf{k}}|\boldsymbol{\nabla}_{\mathbf{k}}g_{\alpha\mathbf{k}}\rangle\,, \tag{84}
$$

$$
\langle\mathbf{i}\alpha|\,\mathbf{r}\,|\mathbf{0}\beta\rangle = \int_\Omega d^2\mathbf{r}\,w^*_\alpha(\mathbf{r}-\mathbf{r_i})w_\beta(\mathbf{r})\mathbf{r} = i\frac{A_\Omega}{(2\pi)^2}\int_{B.Z.}d^2\mathbf{k}\,e^{i\mathbf{k}\cdot\mathbf{r_i}}\langle g_{\alpha\mathbf{k}}|\boldsymbol{\nabla}_{\mathbf{k}}g_{\beta\mathbf{k}}\rangle\,. \tag{85}
$$

A_Ω is the area of the unit cell, and the periodic Bloch functions indexed by α instead of n are obtained by the transformation $g_{\alpha\mathbf{k}} = \sum_n [U_{\mathbf{k}}]_{\alpha,n}g_{n\mathbf{k}}$. We have also defined the ket $|\boldsymbol{\nabla}_{\mathbf{k}}g_{\alpha\mathbf{k}}\rangle$ as $\langle\mathbf{r}|\boldsymbol{\nabla}_{\mathbf{k}}g_{\alpha\mathbf{k}}\rangle = \boldsymbol{\nabla}_{\mathbf{k}}g_{\alpha\mathbf{k}}(\mathbf{r})$ in the identities (84)-(85) which are derived in Ref. 6. The above functional can obviously be interpreted as the sum of the spreads of the Wannier functions. The localization functional can be split into two contributions

$$
F = F_I + \widetilde{F}\,, \tag{86}
$$

$$
F_I = \sum_\alpha\left[\langle\mathbf{0}\alpha|\,r^2\,|\mathbf{0}\alpha\rangle - \sum_{\mathbf{i}\beta}|\langle\mathbf{i}\beta|\,\mathbf{r}\,|\mathbf{0}\alpha\rangle|^2\right]\,, \tag{87}
$$

$$
\widetilde{F} = \sum_{\substack{\mathbf{i}\beta\\ \mathbf{i}\beta\neq\mathbf{0}\alpha}}|\langle\mathbf{i}\beta|\,\mathbf{r}\,|\mathbf{0}\alpha\rangle|^2\,. \tag{88}
$$

The first contribution F_I is gauge invariant in the sense that it is independent of the unitary matrix $U_{\mathbf{k}}$ and it is interesting to provide an expression for F_I in terms of the Bloch functions $g_{\alpha\mathbf{k}}$ using the identities (84)-(85)

$$\begin{aligned}
F_I = &\sum_i \sum_\alpha \Bigg[\frac{A_\Omega}{(2\pi)^2}\int_{B.Z.} d^2\mathbf{k}\,\langle \partial_{k_i} g_{\alpha\mathbf{k}} | \partial_{k_i} g_{\alpha\mathbf{k}}\rangle \\
&-\sum_{\mathbf{i}\beta}\left(\frac{A_\Omega}{(2\pi)^2}\right)^2 \int_{B.Z.} d^2\mathbf{k}_1 \int_{B.Z.} d^2\mathbf{k}_2\, e^{i(\mathbf{k}_1-\mathbf{k}_2)\cdot\mathbf{r}_\mathbf{i}}\, \langle \partial_{k_i} g_{\alpha\mathbf{k}_2} | g_{\beta\mathbf{k}_2}\rangle\, \langle g_{\beta\mathbf{k}_1} | \partial_{k_i} g_{\alpha\mathbf{k}_1}\rangle \Bigg] \\
&= \frac{A_\Omega}{(2\pi)^2}\sum_i \int_{B.Z.} d^2\mathbf{k} \sum_\alpha \Bigg[\langle \partial_{k_i} g_{\alpha\mathbf{k}} | \partial_{k_i} g_{\alpha\mathbf{k}}\rangle + \sum_\beta \langle g_{\alpha\mathbf{k}} | \partial_{k_i} g_{\beta\mathbf{k}}\rangle \langle g_{\beta\mathbf{k}} | \partial_{k_i} g_{\alpha\mathbf{k}}\rangle \Bigg] \\
&= \frac{A_\Omega}{(2\pi)^2}\sum_i \int_{B.Z.} d^2\mathbf{k}\, \left[\mathrm{Tr}[(\partial_i \bar{\mathcal{G}}_{\mathbf{k}}^\dagger)(\partial_i \bar{\mathcal{G}}_{\mathbf{k}})] + \mathrm{Tr}[\bar{\mathcal{G}}_{\mathbf{k}}^\dagger\,(\partial_i \bar{\mathcal{G}}_{\mathbf{k}})\,\bar{\mathcal{G}}_{\mathbf{k}}^\dagger\,(\partial_i \bar{\mathcal{G}}_{\mathbf{k}})]\right] \\
&= \frac{A_\Omega}{(2\pi)^2}\sum_i \int_{B.Z.} d^2\mathbf{k}\,\frac{1}{2}\mathcal{B}_{ii}(\mathbf{k}) = \frac{A_\Omega}{2\pi}\,\frac{1}{2}\mathrm{Tr}[\mathcal{M}]\,.
\end{aligned} \tag{89}$$

In the last line we have used the definition of quantum geometric tensor $\mathcal{B}_{ij}(\mathbf{k})$ and of the invariant matrix $\mathcal{M}$ [see Eq. (68)]. In particular the matrix $[\bar{\mathcal{G}}_{\mathbf{k}}]_{\mathbf{r},\alpha} = g_{\alpha\mathbf{k}}(\mathbf{r})$ is analogous to the matrix $\bar{\mathcal{G}}_{\mathbf{k}}$ as defined in Eq. (69) and used in the main text with the only difference that the index $\mathbf{r}$ is continuous and has to been integrated on the whole unit cell when the matrix product is performed. Using the arithmetic mean-geometric mean inequality $(\lambda_1+\lambda_2)/2 \geq \sqrt{\lambda_1\lambda_2}$ for the eigenvalues of the positive matrix $\mathcal{M}$ and indicating with $\mathcal{M}^{\mathrm{R}}$ the real part of $\mathcal{M}$ and with $\mathcal{M}^{\mathrm{I}}$ the imaginary part, one has the following chain of equalities/inequalities

$$\frac{1}{2}\mathrm{Tr}[\mathcal{M}] = \frac{1}{2}\mathrm{Tr}[\mathcal{M}^{\mathrm{R}}] \geq \sqrt{\det[\mathcal{M}^{\mathrm{R}}]} \geq \sqrt{\det[\mathcal{M}^{\mathrm{I}}]} = |C|\,. \tag{90}$$

The second inequality is Eq. (23) in the main text. Thus the Chern number of the composite band provides a bound from below of the localization functional, namely the result stated in the main text

$$F \geq \frac{A_\Omega}{2\pi}|C|\,. \tag{91}$$

Supplementary Note 6: Berezinsky-Kosterlitz-Thouless transition

The low energy fluctuations of the order parameter phase $\theta(\mathbf{r})$ have an energy cost given by the Hamiltonian of the classical 2D XY model

$$\mathcal{H}_{\mathrm{XY}} = \frac{\Upsilon}{2}\int d^2\mathbf{r}\,\left(\boldsymbol{\nabla}\theta(\mathbf{r})\right)^2\,. \tag{92}$$

The "stiffness" Υ is in fact related to the superfluid weight by the relation $\Upsilon = D_{\mathrm{s}}\hbar^2/4$ where the factor 4 that appears in this last equation is due to the fact that in our notation $\boldsymbol{\nabla}\theta(\mathbf{r}) = 2\mathbf{q}$ for a constant phase gradient. At the Berezinsky-Kosterlitz-Thouless transition temperature T_{BKT} the vortex unbinding due to thermal fluctuations leads to the destruction of the phase order and thus of superfluidity. The BKT critical temperature can be obtained by the universal relation[7] $\Upsilon(T_{\mathrm{BKT}})/(k_{\mathrm{B}}T_{\mathrm{BKT}}) = 2/\pi$. We would like to estimate the BKT critical temperature with the finite temperature result obtained for the superfluid weight (67). For simplicity only the half filling case $\nu = 1/2$ is considered. The zero temperature value of the energy gap $\Delta(T=0) = Un_\phi/2 = 2k_{\mathrm{B}}T_{\mathrm{c}}$ is taken as the energy scale. The temperature-dependence of the energy gap is then obtained by the equation (compare with Eq. (61) and (64))

$$\frac{\Delta(T)}{\Delta(0)} = \tanh\left(\frac{T_{\mathrm{c}}}{T}\frac{\Delta(T)}{\Delta(0)}\right)\,, \tag{93}$$

while the stiffness is, from Eq. (67),

$$\frac{\Upsilon_{\bar{n}}(T)}{\Delta(0)} = \frac{2\bar{n}+1}{4\pi}\frac{\Delta^2(T)}{\Delta^2(0)} \,. \tag{94}$$

The dependence of the stiffness on the Landau level number $\bar{n}$ has been introduced. Then the equation that needs to be solved in order to find the BKT critical temperature is

$$\frac{2\bar{n}+1}{4}\frac{\Delta^2(T_{\mathrm{BKT}})}{\Delta^2(0)} = \frac{T_{\mathrm{BKT}}}{T_{\mathrm{c}}} \,. \tag{95}$$

Eq. (93) and (95) can be solved numerically and the result is shown in Supplementary Figure 1. The BKT temperatures for $\bar{n} = 0, 1, 2$ are approximately $T_{\mathrm{BKT}} \approx 0.25,\ 0.61,\ 0.75\,T_{\mathrm{c}}$, respectively. This justifies our assertion in the main text that the mean-field temperature is in fact a good estimate of the real temperature of the superconducting transition in the lowest bands of the Harper model.

Supplementary References

1. Schnyder, A. P., Ryu, S., Furusaki, A. & Ludwig, A. W. W., Classification of topological insulators and superconductors in three spatial dimensions, Phys. Rev. B **78**, 195125 (2008).

2. Taylor, E., Griffin, A., Fukushima, N. & Ohashi, Y., Pairing fluctuations and the superfluid density through the BCS-BEC crossover, Phys. Rev. A **74**, 063626 (2006).

3. Grosso G., & Pastori Parravicini, G., *Solid State Physics*, 2nd ed., Elsevier (2014).

4. Brouder, C., Panati, G., Calandra, M., Mourougane, C. & Marzari, N., Exponential Localization of Wannier Functions in Insulators, Phys. Rev. Lett. **98**, 046402 (2007).

5. Harper, F., Simon, S. H. & Roy, R., Perturbative approach to flat Chern bands in the Hofstadter model, Phys. Rev. B **90**, 075104 (2014).

6. Marzari, N., & Vanderbilt, D., Maximally localized generalized Wannier functions for composite energy bands, Phys. Rev. B **56**, 12847-12865 (1997).

7. Nelson, D. R. & Kosterlitz, J. M., Universal Jump in the Superfluid Density of Two-Dimensional Superfluids, Phys. Rev. Lett. **39**, 1201-1204 (1977).
